# Fetal alcohol spectrum disorder predisposes to metabolic abnormalities in adulthood

**DOI:** 10.1172/JCI132139

**Published:** 2020-03-23

**Authors:** Olivia Weeks, Gabriel D. Bossé, Isaac M. Oderberg, Sebastian Akle, Yariv Houvras, Paul J. Wrighton, Kyle LaBella, Isabelle Iversen, Sahar Tavakoli, Isaac Adatto, Arkadi Schwartz, Daan Kloosterman, Allison Tsomides, Michael E. Charness, Randall T. Peterson, Matthew L. Steinhauser, Pouneh K. Fazeli, Wolfram Goessling

**Affiliations:** 1 Division of Genetics, Brigham and Women’s Hospital, Harvard Medical School, Boston, Massachusetts, USA.; 2 Department of Pharmacology and Toxicology, College of Pharmacy, University of Utah, Salt Lake City, Utah, USA.; 3 Organismic and Evolutionary Biology, Harvard University, Cambridge, Massachusetts, USA.; 4 Department of Surgery and; 5 Department of Medicine, Weill Cornell Medical College, New York, New York, USA.; 6 Department of Stem Cell and Regenerative Biology, Harvard University, Cambridge, Massachusetts, USA.; 7 Veterans Affairs Boston Healthcare System, West Roxbury, Massachusetts, USA.; 8 Neurology, Harvard Medical School, Boston, Massachusetts, USA.; 9 Department of Neurology, Boston University School of Medicine, Boston, Massachusetts, USA.; 10 Division of Cardiovascular Medicine, Department of Medicine, Brigham and Women’s Hospital, Boston, Massachusetts, USA.; 11 Broad Institute, Massachusetts Institute of Technology and Harvard University, Cambridge, Massachusetts, USA.; 12 Neuroendocrine Unit, Massachusetts General Hospital, Harvard Medical School, Boston, Massachusetts, USA.; 13 Harvard Stem Cell Institute, Cambridge, Massachusetts, USA.; 14 Dana-Farber Cancer Institute, Boston, Massachusetts, USA.; 15 Harvard-MIT Division of Health Sciences and Technology, Cambridge, Massachusetts, USA.; 16 Division of Gastroenterology, Massachusetts General Hospital, Boston, Massachusetts, USA.

**Keywords:** Development, Metabolism, Embryonic development, Obesity

## Abstract

Prenatal alcohol exposure (PAE) affects at least 10% of newborns globally and leads to the development of fetal alcohol spectrum disorders (FASDs). Despite its high incidence, there is no consensus on the implications of PAE on metabolic disease risk in adults. Here, we describe a cohort of adults with FASDs that had an increased incidence of metabolic abnormalities, including type 2 diabetes, low HDL, high triglycerides, and female-specific overweight and obesity. Using a zebrafish model for PAE, we performed population studies to elucidate the metabolic disease seen in the clinical cohort. Embryonic alcohol exposure (EAE) in male zebrafish increased the propensity for diet-induced obesity and fasting hyperglycemia in adulthood. We identified several consequences of EAE that may contribute to these phenotypes, including a reduction in adult locomotor activity, alterations in visceral adipose tissue and hepatic development, and persistent diet-responsive transcriptional changes. Taken together, our findings define metabolic vulnerabilities due to EAE and provide evidence that behavioral changes and primary organ dysfunction contribute to resultant metabolic abnormalities.

## Graphical abstract

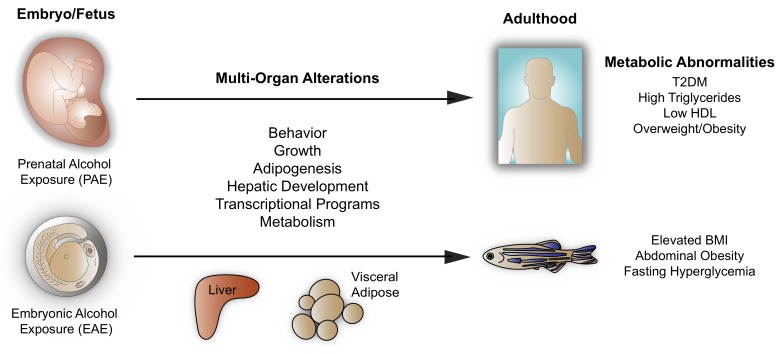

## Introduction

Alcohol and its primary metabolite, acetaldehyde, are teratogens, and exposure during gestation detrimentally affects fetal development (1–3). More than 10% of pregnant women worldwide consume alcohol, and recent estimates suggest that 1% to 5% of US school-age children have fetal alcohol spectrum disorders (FASDs) (4–7). FASD is an umbrella term describing a group of clinical conditions resulting from prenatal alcohol exposure (PAE), including fetal alcohol syndrome (FAS), partial fetal alcohol syndrome (pFAS), alcohol-related neurodevelopmental disorder (ARND), and alcohol-related birth defects (ARBD) (8). FASD patients have variable features, including facial dysmorphology, microcephaly, cognitive and behavioral deficits, and organ malformations (7, 9–11). The physiological effects of PAE are thought to last a lifetime; however, the metabolic health outcomes of FASDs on patients are not well understood (12). In particular, the occurrence of metabolic syndrome, obesity, and type 2 diabetes mellitus (T2DM) in adults with FASDs are unknown.

According to the developmental origins of health and disease hypothesis, environmental factors present during prenatal stages can alter an individual’s response to stress later in life (13). Adverse intrauterine events have been repeatedly associated with metabolic, endocrine, and cardiovascular disorders (13–17). Human studies suggest that children and adolescents with FASDs have increased body fat, a higher incidence of obesity, and hypertension (18, 19). However, despite additional reports using animal models suggesting that perinatal and gestational alcohol exposure are connected to poor metabolic health outcomes, there are multiple studies indicating that PAE is not associated with increased fat mass or metabolic syndrome following specific alcohol exposure windows (12, 18, 20–30). These conflicting reports highlight the need to clarify which adverse health outcomes are associated with PAE and to define disease mechanisms.

This study sought to determine whether PAE increases the risk of adult obesity and metabolic disease in human and zebrafish cohorts. We demonstrate that adult patients with any FASD diagnosis have an increased incidence of T2DM, low HDL, and high triglyceride levels relative to matched controls. Males from the FASD cohort had a higher incidence of these metabolic abnormalities despite a lower BMI, whereas females had a higher incidence of overweight and obesity. To address limitations posed by these human studies, we developed an adult zebrafish FASD model and performed controlled population studies to assess the metabolic sequelae of embryonic alcohol exposure (EAE). In zebrafish, EAE predisposed to metabolic abnormalities, including diet-induced visceral adiposity, elevated BMI, and fasting hyperglycemia. Our study pinpoints FASDs as a risk factor for metabolic disease and identifies developmental, behavioral, and molecular regulators of this outcome.

## Results

### FASDs are associated with multiple metabolic abnormalities.

**Figure 1:**
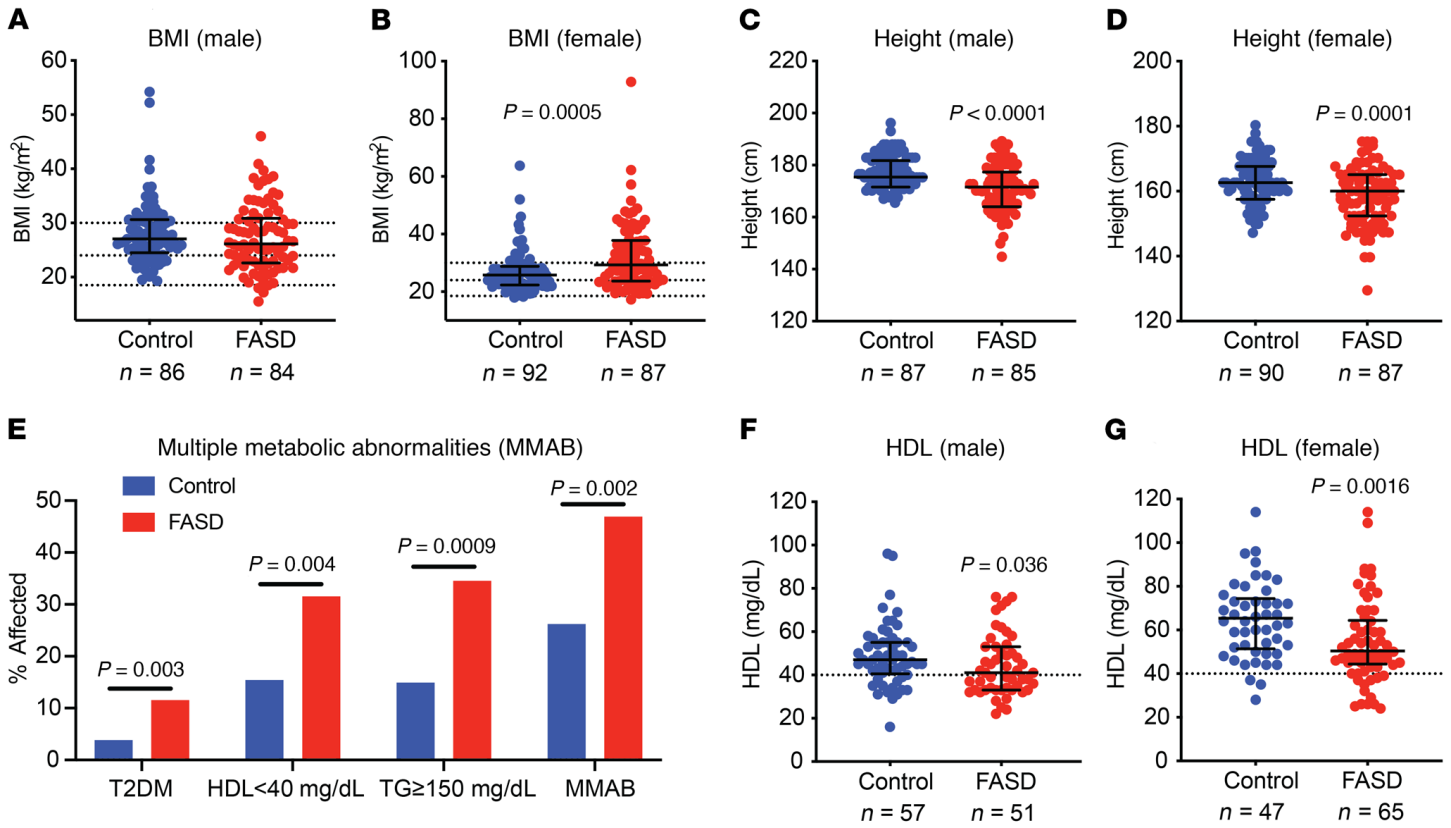
FASD patients have an increased incidence of metabolic abnormalities. (**A** and **B**) BMI distribution of male (*P* = 0.26) and female (*P* = 0.0005) control and FASD cohorts. Wilcoxon’s test. (**C** and **D**) Height distribution of male (*P* < 0.0001, Wilcoxon’s test) and female (*P* = 0.0001, Student’s unpaired *t* test) cohorts. (**E**) Incidence of multiple metabolic abnormalities in combined male and female cohorts. T2DM (*P* = 0.003, logistic regression), HDL (*P* = 0.004, Pearson’s χ^2^ test), TG (*P* = 0.0009, Pearson’s χ^2^ test), multiple metabolic abnormalities (*P* = 0.002, Pearson’s χ^2^ test). (**F** and **G**) HDL measurements in male (*P* = 0.035) and female (*P* = 0.0016) cohorts. Wilcoxon’s test. Data represent median with interquartile range. Sample numbers (*n*) noted under figure panels.

To test the hypothesis that PAE predisposes to cardiometabolic disease, we performed a retrospective cross-sectional study examining the incidence of metrics of cardiometabolic health in adults with any FASD diagnosis, including FAS, pFAS, ARND, and ARBD. Using the patient database registry at a large academic health system (Research Patient Data registry [RPDR] of Partners HealthCare System), we identified male and female patients 18 years or older with FASDs (*n* = 208) and controls matched for age, sex, and race/ethnicity (*n* = 208). The median (interquartile range) age of the entire cohort was 29.7 years (22.2 to 44.4) (Supplemental Figure 1, A and B, and Table 1; supplemental material available online with this article; https://doi.org/10.1172/JCI132139DS1). Of those in the FASD cohort, 26.9% were overweight (25 ≤ BMI < 30 kg/m^2^), 38.0% obese (BMI ≥ 30 kg/m^2^), and 2.9% underweight (BMI ≤ 18.5 kg/m^2^; Table 1). FASD was a risk factor for an overweight/obese phenotype in females (69.0% of FASD females versus 54.3% of controls, *P* = 0.04) but not males (60.7% of FASD males versus 73.3% of controls, *P* = 0.08) (Table 1 and Figure 1, A and B). Instead, male FASD patients were more likely to be underweight (*P* = 0.04; Table 1). Furthermore, FASD patients were significantly shorter than controls; patients had a mean height of 164.9 ± 0.9 cm (SEM) compared with a mean height of 170.4 ± 0.7 cm in controls (*P* < 0.0001; Table 1). This height difference was observed in both male FASD patients (mean height of male FASD cohort: 171.6 ± 1.0 cm versus mean height of male controls 177.9 ± 0.7 cm, *P* < 0.0001) and female FASD patients (mean height of female FASD cohort: 158.4 ± 1.0 cm versus mean height of female controls: 163.2 ± 0.7, *P* = 0.0001; Table 1 and Figure 1, C and D). In fact, the male FASD cohort’s mean height was approximately 2 inches below the US average for males aged 20 years or more (31).

Significantly more FASD patients had T2DM (11.5% versus 3.8% of controls, unadjusted *P* value = 0.003; Table 1, Figure 1E, and Supplemental Figure 1, C and D). In the male FASD cohort, the difference in T2DM was significant after controlling for BMI (*P* = 0.03), but not in the female cohort (Table 1). In females, 11.4% of the FASD cohort had T2DM versus 2.9% of controls (unadjusted *P* value: 0.02), but this difference was no longer significant after controlling for BMI (*P* = 0.16), suggesting that this difference in prevalence of T2DM was predominantly mediated by BMI in the female cohort (Table 1). Significantly more FASD patients had low HDL (<40 mg/dL) and elevated triglycerides (≥150 mg/dL; Table 1; Figure 1, E–G; and Supplemental Figure 1, E and F). Of the FASD cohort, 31.9% had low HDL versus 15.4% of controls (*P* = 0.004), and 34.5% of the FASD cohort versus 14.9% controls (*P* = 0.0009) had elevated triglycerides (Table 1 and Figure 1, E–G). Since this study is a retrospective analysis, not all patients had the same tests performed, resulting in a different number of patients for each parameter examined, but in 216 individuals (*n* = 103 controls and *n* = 113 FASD patients), we could accurately assess whether subjects had 2 or more metabolic abnormalities (overweight/obese BMI, T2DM, HDL <40 mg/dL, and/or triglycerides ≥150 mg/dL). FASD subjects were significantly more likely to have 2 or more metabolic abnormalities (46.9% in FASDs versus 26.2% in controls, *P* = 0.002) (Table 1 and Figure 1E). These findings reveal PAE as a risk factor for developing features of the metabolic syndrome in adulthood, independently of BMI in the case of the male cohort.

### EAE potentiates BMI gains and hyperglycemia in adult male zebrafish.

**Figure 2:**
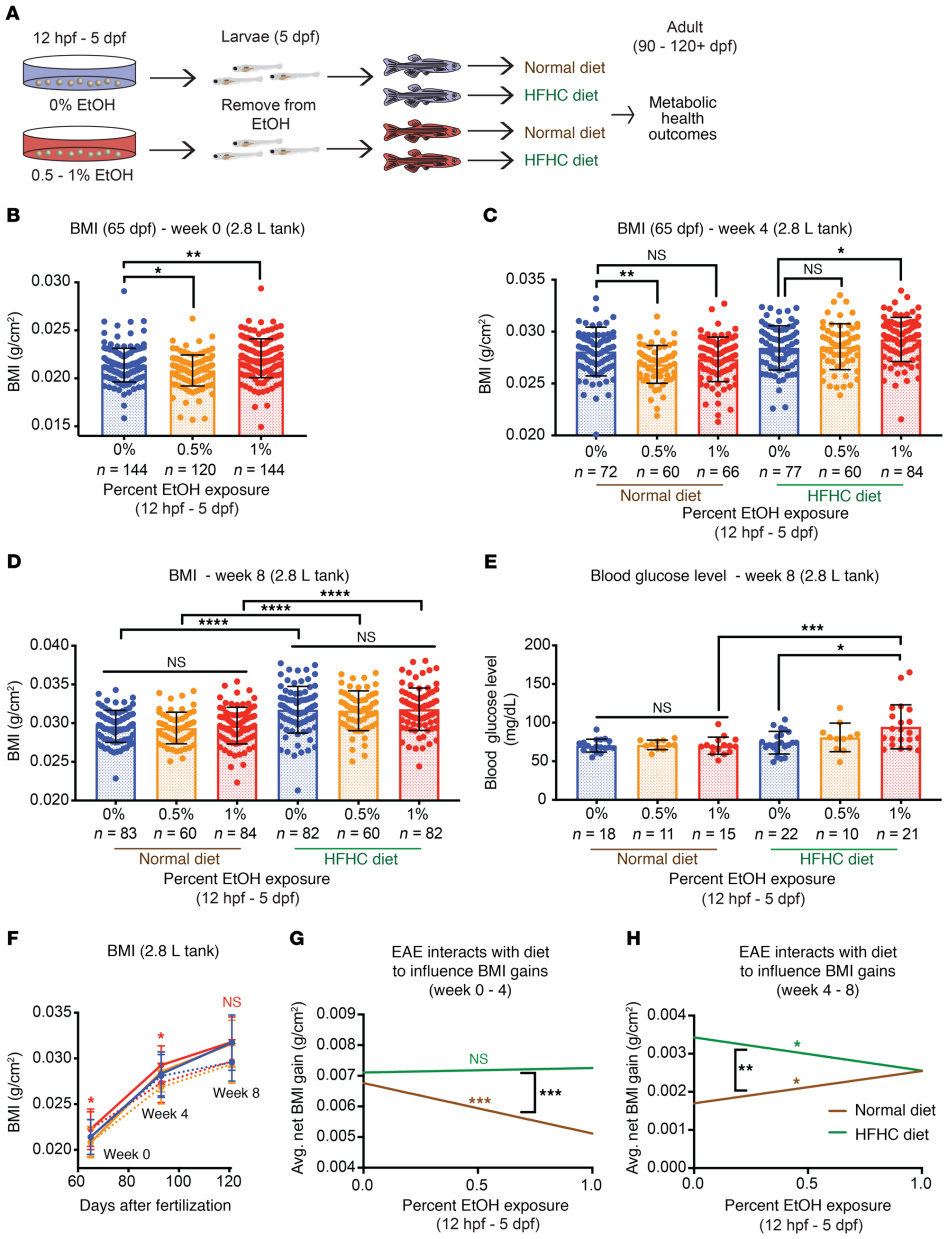
EAE alters growth dynamics and serves as a risk factor for metabolic disease in adult males. (**A**) Protocol for assessing metabolic health outcomes following EAE. (**B**) BMI at the initiation of the confirmatory diet challenge. 1% EtOH–exposed males have a significantly higher BMI than controls. *P = 0.0193; ** *P* = 0.005, Brown-Forsythe and Welch’s ANOVA with Games-Howell multiple comparisons test. (**C**) BMI following 4 weeks of diet challenge. 1% EtOH–exposed males on HFHC but not ND have a larger BMI than matched controls. **P = 0.002; *P = 0.0429, ordinary 1-way ANOVA with Tukey’s multiple comparisons test. (**D**) After 8 weeks of diet challenge, EAE has no effect on BMI in either diet group. HFHC diet increases BMI in all cohorts. ****P_adj_ ≤ 0.0001, 2-way ANOVA with Šidák’s multiple comparisons test. (**E**) Fasting BG level after 8 weeks of diet. *P = 0.0181, Brown-Forsthye and Welch’s ANOVA with Dunnett’s T3 multiple comparisons test; ***P = 0.0006, 2-way ANOVA with Šidák’s multiple comparisons test. Error bars show mean with SD. (**F**) Time course of BMI in 0% EtOH (blue), 0.5% EtOH (orange), and 1% EtOH (red) cohorts receiving ND (dotted line) or HFHC diet (solid line). *P < 0.05, ordinary 1-way ANOVA with Tukey’s multiple comparisons test. (**G** and **H**) Ethanol interacts with diet to influence BMI gain for weeks 0 to 4 (ND: slope = –0.0016, *P* = 2.15 × 10^–06^; interaction [difference in slope]: 0.0017752, *P* = 0.000317) and weeks 4 to 8 of the diet (ND: slope = 0.00084, *P* = 0.0255; HFHC diet: slope = –0.00087, *P* = 0.0225; interaction: –0.0017148; *P* = 0.00146). The units for slope and interaction are (g/cm^2^)/ethanol%. *P* values determined using linear regression. Error bars show mean with SD. Sample numbers (*n*) noted under figure panels.

To clarify the connection between metabolic health and PAE, we performed population studies in AB strain zebrafish and evaluated body length, weight, BMI, fasting blood glucose (BG) levels, and adiposity. Sibling-matched cohorts were exposed to 0%, 0.5%, or 1% EtOH during embryogenesis (12 hours post fertilization [hpf] to 5 days post fertilization [dpf]); raised in the absence of ethanol until late juvenile stages (60–65 dpf); and challenged with a high-fat, high-cholesterol (HFHC) or normal diet (ND) for 4 or 8 weeks into adulthood (90–120 dpf; Figure 2A). Treatment with 0.5% to 1% EtOH can sufficiently induce physiologically relevant tissue EtOH concentrations, and 12 hpf–5 dpf covers the period of zebrafish organogenesis (32, 33).

We first evaluated the impact of EAE on embryonic growth. Immediately following EAE, AB strain larvae displayed mild growth restriction, consistent with the short stature phenotypes typical of human FAS (Supplemental Figure 2, A–C, and ref. 34). When removed from ethanol, larvae exhibited compensatory growth and reached the same size as control-matched siblings by 20 dpf (Supplemental Figure 2D). EAE had no significant impact on body length, weight, or BMI in sexually immature juveniles (34 dpf; Supplemental Figure 2, E–G). Next, we conducted a pilot study to assess diet-induced obesity risk in male and female cohorts housed in 6-L tanks. At the start of the diet challenge (60 dpf), neither EAE females nor males had significant differences in BMI relative to controls (Supplemental Figure 3, A and B). Females exposed to 0.5% EtOH had a significantly reduced BMI relative to females expose to 1% EtOH, indicating that EtOH exposure level may result in subtle differences in BMI (Supplemental Figure 3B). After 4 weeks of ND challenge, neither EAE males nor females had a significantly higher BMI than sex-matched control siblings (Supplemental Figure 3, C and D). However, after 4 weeks of HFHC diet challenge, males but not females exposed to 1% EtOH developed a significantly elevated BMI (Supplemental Figure 3, C–E). These findings suggested that EAE is a risk factor for diet-induced obesity in male zebrafish.

We next confirmed that EAE is a risk factor for increased BMI in males using separate cohorts housed in 2.8-L tanks. At 65 dpf, 1% EtOH–exposed males had a significantly elevated BMI at the initiation of the diet challenge (65 dpf), which resulted from an increase in body weight (Figure 2B and Supplemental Figure 3, F and G). In contrast, females, in an independent study (2.8-L tanks), did not have significantly increased BMI during late juvenile stages (60–70 dpf; Supplemental Figure 4A). After 4 weeks of diet challenge, 1% EtOH males receiving a HFHC diet, but not ND, maintained significant elevations in BMI relative to controls (Figure 2C). Increasing EAE levels positively correlated with BMI with HFHC diet (*P* = 0.0074) and negatively correlated with BMI with ND (*P* = 0.02; Supplemental Figure 4B). Importantly, males from the 0.5% EtOH cohort had a significantly lower body weight and length under ND conditions relative to controls (Supplemental Figure 4, C and D). Collectively, these studies demonstrate that EAE is a risk factor for diet-induced BMI elevations during adulthood and that this phenotype is consistent regardless of tank size. Since the HFHC diet challenge includes excess calories, fat, and cholesterol, additional studies are needed to clarify the relevant impact of each of these dietary components on excess BMI gain.

Although there were initial elevations in BMI in the EAE cohorts, after a total of 8 weeks of diet challenge, control and EAE siblings achieved the same BMI, length, and weight (Figure 2D and Supplemental Figure 4, E and F). Despite achieving the same BMI, 1% EtOH–exposed (12 hpf–5 dpf) adults receiving a HFHC diet had a more rapid progression toward fasting hyperglycemia. Males exposed to 1% EtOH fed the HFHC diet, but not ND, developed significant increases in fasting BG levels relative to family-matched sibling controls (Figure 2E). Consistent with the human cohort, these data suggest that EAE increases the risk for short-term BMI gains and raises the risk for impaired glucose tolerance, even in the context of equivalent BMIs.

### The adaptive response to HFHC feeding is altered by EAE.

The dynamics of BMI gain in response to normal and HFHC diet differed between control and EAE adults, revealing that EAE adults have an altered adaptive response to nutrient intake (Figure 2F). Under ND conditions, increasing EtOH concentrations negatively correlated with net BMI gain for the first 4 weeks of diet challenge (*P* = 2.15 × 10^–06^; Figure 2, G and H, and Supplemental Figure 4G). However, from weeks 4 to 8, increasing ethanol concentration positively correlated with net BMI gain (*P* = 0.0255; Figure 2, G and H, and Supplemental Figure 4G). This suggests that under ND conditions, EAE adults initially have a slower BMI gain than controls but increase their growth for the remaining weeks. A reverse pattern of BMI gain was observed for the HFHC diet condition. EAE fish had slight but nonsignificant increases in BMI gain relative to controls for weeks 0 to 4, but then experienced significantly reduced BMI gains for weeks 4 to 8 of the challenge (*P* = 0.0225; Figure 2, G and H, and Supplemental Figure 4H). Although the week 0 to 4 time point did not show significant increases in net BMI gain relative to controls, in a follow-up experiment with EAE fish housed in 1.4-L tanks, HFHC diet resulted in significantly increased BMI gains over weeks 0 to 4 (Supplemental Figure 4I). Despite the fact that 0%, 0.5%, and 1% EtOH–exposed (12 hpf–5 dpf) cohorts gained the same amount of body mass over the 8-week period, the periods when BMI gain was achieved were significantly affected by the interaction between prior EAE and diet (Figure 2, G and H).

### Visceral adiposity is preferentially increased in EAE adults.

**Figure 3:**
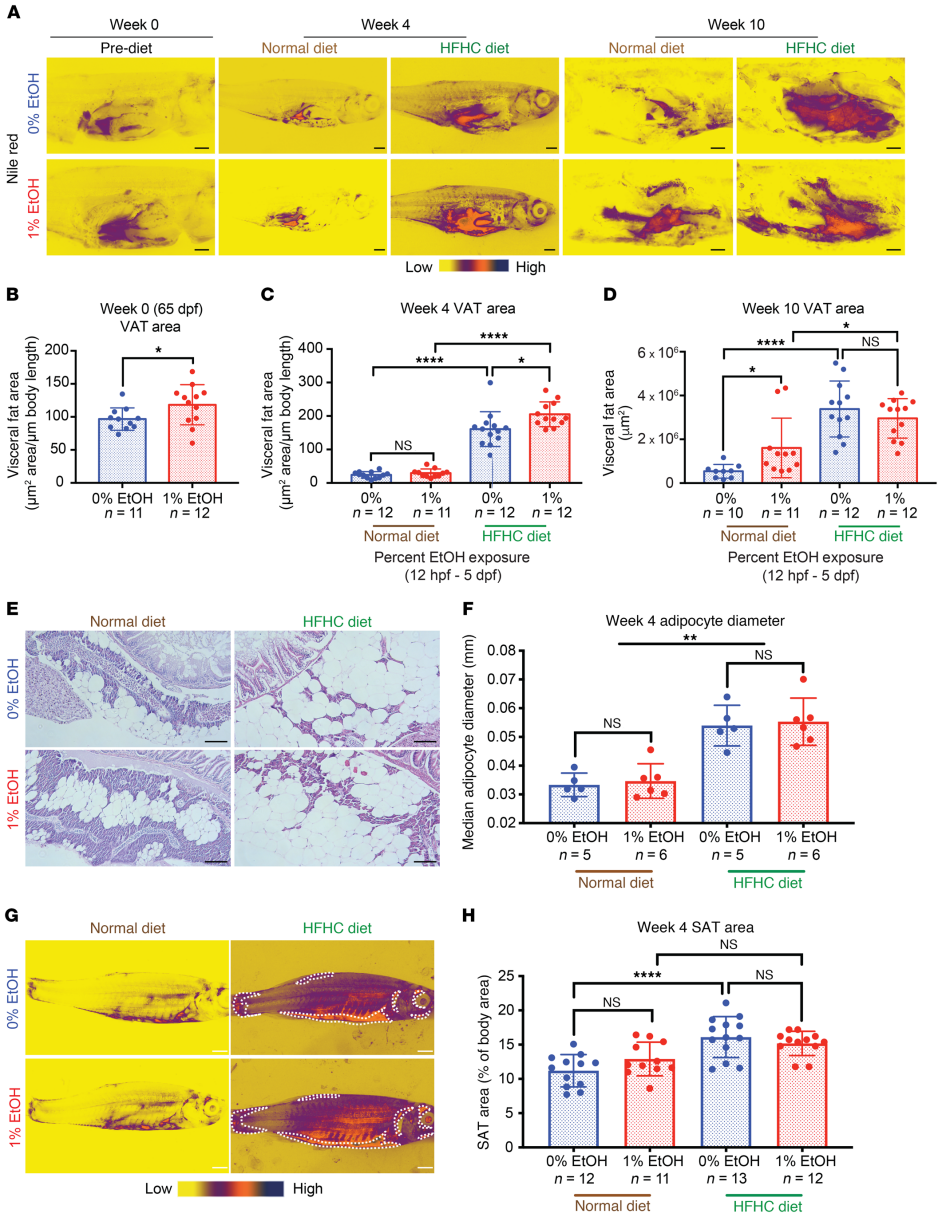
EAE is a risk factor for increased visceral adiposity in adulthood. (**A**) Nile red staining of internal organs and VAT visualized after skin and body wall muscle removal. Scale bars: 1 mm. (**B**) Quantification of VAT area before diet challenge. 1% EtOH–exposed (12 hpf–5 dpf) males have a larger VAT depot than controls at 65 dpf. *P = 0.0497, Student’s unpaired 2-tailed *t* test. (**C**) Quantification of VAT area after 4 weeks of diet challenge. EAE adults have a larger VAT than controls in response to HFHC. *P = 0.0103; ****P < 0.0001, 2-way ANOVA with Tukey’s multiple comparisons test. (**D**) Quantification of VAT area after 10 weeks of diet challenge. EAE adults receiving the ND have a larger VAT than controls. *P = 0.0102, Student’s 2-tailed unpaired *t* test. HFHC diet induces significant gains in VAT area in both control and EAE adults (*P = 0.0228 [1% EtOH ND versus HFHC], ****P < 0.0001 [0% EtOH ND versus HFHC], 2-way ANOVA Tukey’s multiple comparisons test). (**E**) H&E staining of PVAT following 4 weeks of diet challenge. Scale bars: 0.01 mm. (**F**) Quantification of PVAT adipocyte diameter following normal and HFHC diet. **P = 0.001, 2-way ANOVA. (**G**) Nile red staining of SAT (dotted white outline). Scale bars: 1 mm. Body-wide quantification of SAT was accomplished by combining 2 photos in ImageJ using collage photomerge. (**H**) Quantification of SAT area after 4 weeks of diet challenge. ****P < 0.0001, 2-way ANOVA Tukey’s multiple comparisons test. Error bars show mean with SD. Sample numbers (*n*) noted under figure panels.

Body fat and waist circumference are not routinely assessed in clinical practice, limiting our ability to assess adiposity in the human FASD cohort. Instead, we turned to AB zebrafish and used lipophilic Nile red dye to visualize and quantify adiposity following EAE. Visceral adipose tissue (VAT) size was quantified immediately before and for the duration of the diet challenge (Figure 3A). Males were used because female egg production limits accurate VAT quantification along adult organs. Before the diet challenge, when their BMI was significantly higher than that of controls, 1% EtOH–exposed males had an increased visceral adiposity as visualized by Nile red staining (Figure 3, A and B). This suggests that EAE is a risk factor for increased visceral adiposity during juvenile stages, even without HFHC diet.

After the 4-week diet challenge, adults previously exposed to 1% EtOH maintained a significantly enlarged VAT size relative to controls in the presence of HFHC diet but not ND (Figure 3, A and C). After 8 to 10 weeks of diet challenge, EAE adults receiving the ND had a significantly larger VAT area relative to ND-fed controls, whereas EAE adults receiving the HFHC diet eventually developed the same VAT area as their HFHC diet–fed control siblings (Figure 3, A and D). The significant increase in VAT size in HFHC diet–fed EAE adults was not due to increases in adipocyte cell size (Figure 3, E and F): adipocyte diameter and area significantly correlated with diet treatment, but not with EAE (Figure 3F). This indicates that increased adult VAT size may occur through an increase in white adipocyte number. Subcutaneous adipose tissue (SAT) measurements were unaffected by EAE, demonstrating that the effects on adipose tissue are specific to the VAT compartment (Figure 3, G and H). Collectively, these data indicate that EAE fish have a tendency toward visceral adiposity.

### Adult zebrafish activity is reduced by EAE.

**Figure 4:**
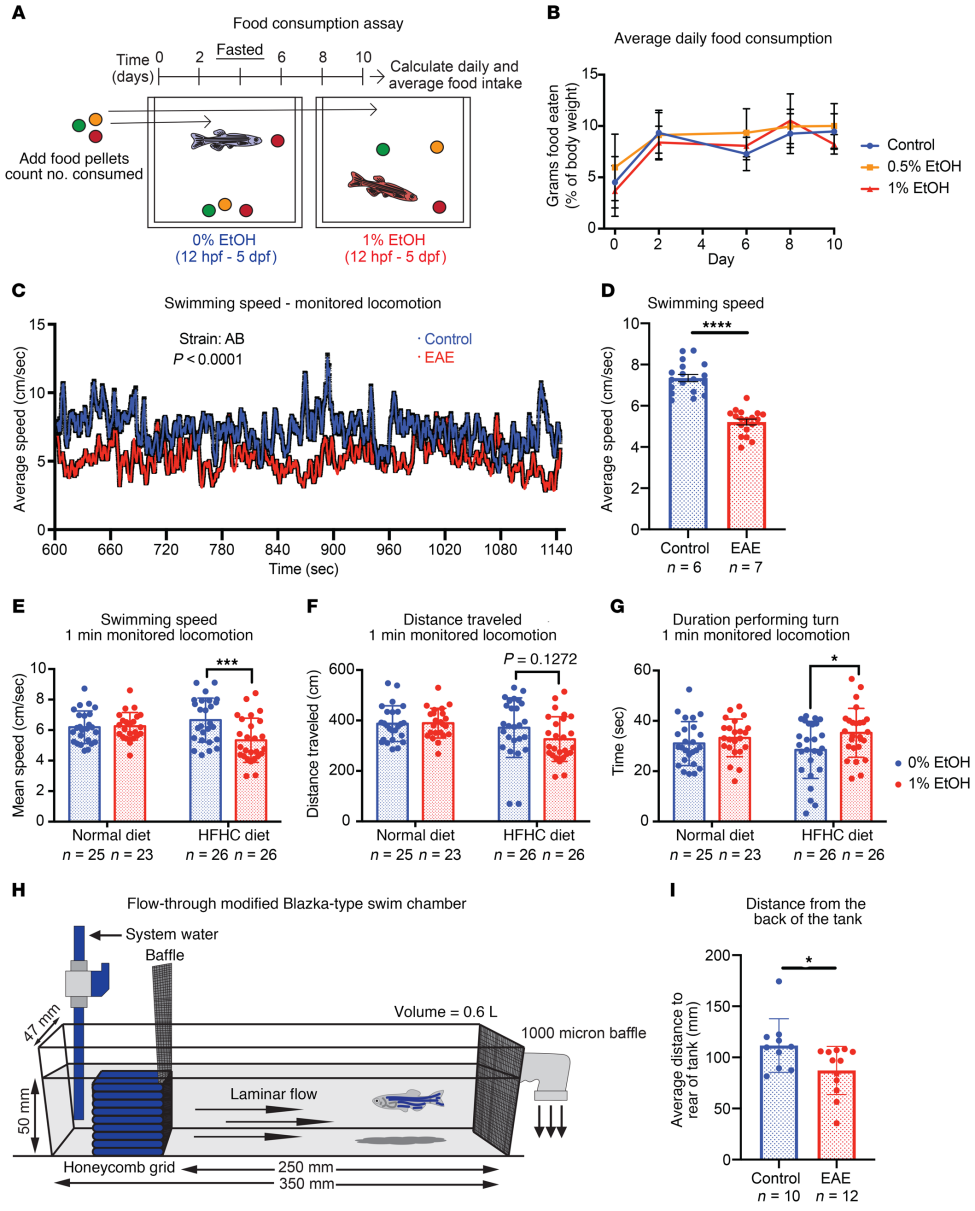
Behavioral assays identify reduced activity level in EAE adults. (**A**) Food consumption assay design. (**B**) EAE has no impact on average daily food consumption in adult males (*n* = 10 animals/group). (**C** and **D**) EAE (1% EtOH) adults have a reduced swimming speed relative to controls (*P* < 0.0001, Student’s unpaired 2-tailed *t* test). (**C**) Average of group speed over 3-second intervals. (**D**) Individual speed averaged over 30-second intervals. ****P < 0.0001. (**E**–**G**) Swimming speed, distance traveled, and duration performing turns in a 1-minute monitored locomotion assay in control and EAE (1% EtOH) adults. EAE cohorts have a normal activity level under ND conditions, but show reduced activity and increased turning behavior with HFHC diet challenge. (**E**) ***P = 0.0005 and (**G**) *P = 0.0227, 2-way ANOVA with Šidák’s multiple comparisons test. (**H** and **I**) EAE adults spend more time near the rear of the tank during a 5-minute laminar flow challenge in a modified Blazka-type swim chamber. *P = 0.0324, Student’s unpaired 2-tailed *t* test. Error bars show mean with SD. Sample numbers (*n*) noted under figure panels.

To identify potential mechanisms of the EAE-associated susceptibility to obesity, we assessed energy balance by monitoring food intake and activity in the AB strain. We measured total food pellet consumption over a 10-day period, normalized to body weight (Figure 4A). EAE had no detectable impact on food consumption in adult males, suggesting that it is an unlikely mechanism for increased adiposity (Figure 4B). Multiple studies have demonstrated that EAE affects zebrafish swimming behaviors (35–38). To determine whether reduced locomotion was present in adults after EAE, we assessed activity level (35, 39–44). Adult fish were subjected to a 10-minute habituation period, followed by a monitored locomotion assay. EAE males had reduced swimming speed and reduced swimming distance, which could result in reduced energy expenditure (Figure 4, C and D). We also performed short 1-minute monitored locomotion assays following a 1-minute habituation period in obese and nonobese zebrafish. In this assay, EAE males receiving a HFHC diet had a significant reduction in swimming speed and spent more time performing turns than controls (Figure 4, E–G). Although this assay duration and habituation period did not detect differences in activity level between nonobese control and EAE fish, the presence of HFHC diet–induced differences suggests that HFHC diet may exacerbate behavioral phenotypes in EAE adults.

Given the apparent propensity for reduced locomotion in EAE adults, we determined whether EAE adults have reduced stamina. Adults were challenged with a laminar flow in a modified Blazka-type swim chamber (Figure 4H). Zebrafish normally swim upstream against the current; however, fatigue or unwillingness to swim can cause the fish to be swept downstream. EAE adults challenged with laminar flow (5 minutes) spent significantly more time near the rear of the chamber than control siblings, indicating that they were unable or unwilling to retain their position in the swim chamber (Figure 4I). Taken together, these findings suggest that mild reductions in activity level and stamina may accelerate the development of diet-induced obesity and poor metabolic health in EAE adults.

### Early alterations in embryonic growth and adipocyte development prime EAE larvae for diet-induced obesity.

**Figure 5:**
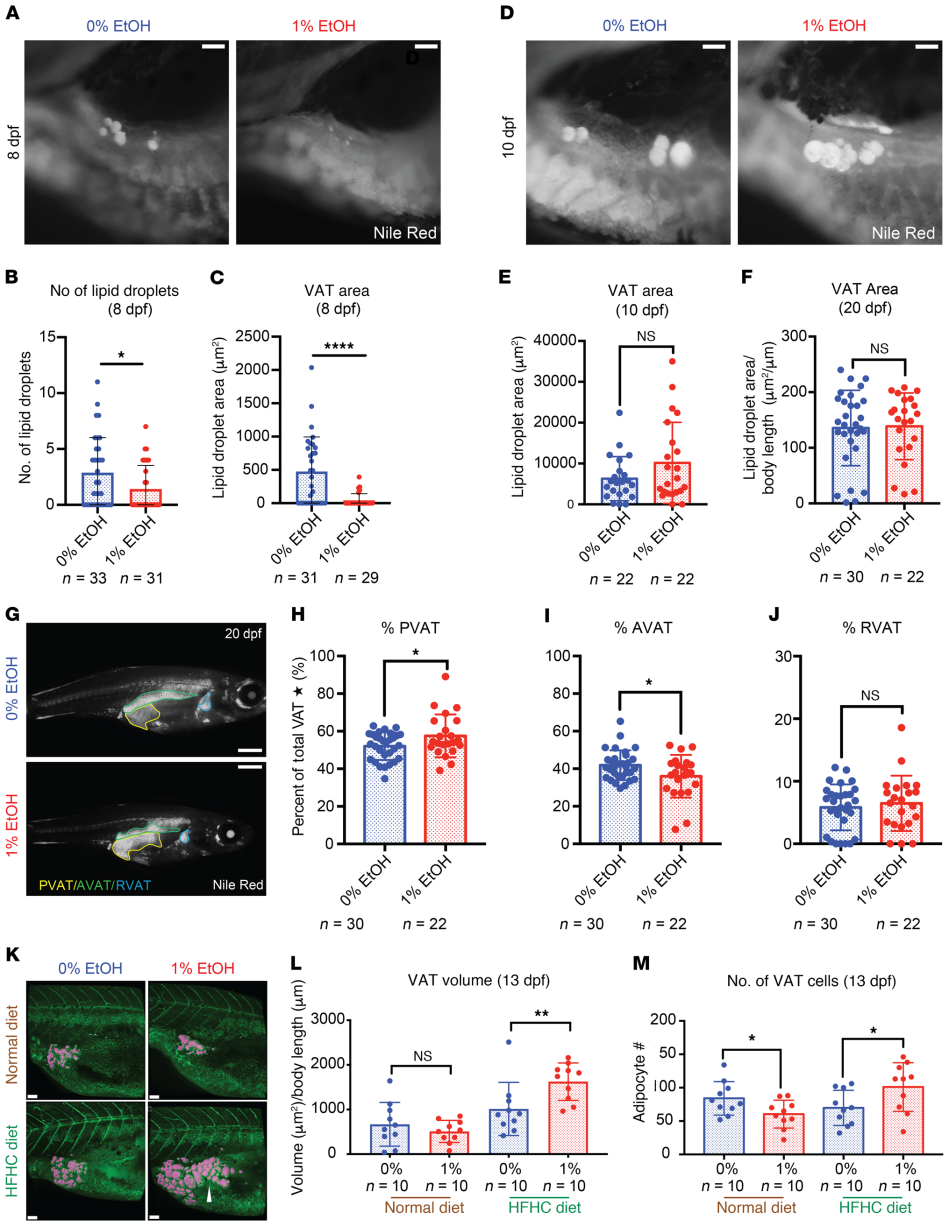
EAE disrupts VAT development and diet-induced VAT gain in larvae. (**A**) EAE delays adipocyte development at 8 dpf as visualized by Nile red staining. Scale bars: 1 mm. (**B**) EAE reduces lipid droplet number at 8 dpf. *P = 0.034, Student’s unpaired 2-tailed *t* test. (**C**) EAE reduces VAT area at 8 dpf. ****P < 0.0001, Student’s unpaired 2-tailed *t* test. (**D**) EAE-treated larvae recover from delayed adipocyte development by 10 dpf as visualized by Nile red staining. Scale bars: 0.1 mm. (**E** and **F**) VAT area recovers by 10 dpf and 20 dpf, and no significant differences in total VAT area are observed. (**G**) VAT distribution, visualized by Nile red staining, is altered in EAE larvae by 20 dpf. Scale bars: 1 mm. (**H**–**J**) EAE larvae have more PVAT than AVAT as a percentage of total VAT area (**H**: *P* = 0.0412; **I**: *P* = 0.0322, Student’s unpaired 2-tailed *t* test; *P < 0.05). RVAT area is not affected by 1% EtOH exposure. Star indicates that total VAT corresponds to RVAT + PVAT + AVAT. (**K**) 3D confocal reconstruction (pink) of Nile red–stained VAT under normal and HFHC diet conditions. Scale bars: 100 μm. (**L**) EAE larvae have a larger VAT volume than controls under HFHC diet but not ND conditions. **P = 0.0096, 2-way ANOVA with Šidák’s multiple comparisons test. (**M**) EAE larvae receiving ND have reduced VAT cell number at 13 dpf (*P* = 0.0345). HFHC diet increases adipocyte number in EAE larvae (*P* = 0.0410). *P < 0.05. Unpaired 2-tailed Student’s *t* test. Error bars show mean with SD. Sample numbers (*n*) noted under figure panels.

Zebrafish first develop white adipose tissue (WAT) around 7 to 8 dpf in the region of the exocrine pancreas (45, 46). Given the fetal growth restriction, we hypothesized that the dynamics of WAT emergence are disrupted by EAE. At 8 dpf, lipid droplet number and total VAT area were significantly reduced in EAE larvae, demonstrating that there are fewer adipocytes (Figure 5, A–C). Despite initial maturation delays, VAT within the developing EAE larvae experienced catch-up growth and the total VAT area reached a normal level by 10 dpf (Figure 5, D–F). Zebrafish have pancreatic VAT (PVAT), abdominal VAT (AVAT), and renal VAT (RVAT) depots that are anatomically distinguishable and readily measurable following lipophilic staining (46). At 13 dpf, no significant differences in VAT distribution were evident (Supplemental Figure 5, A–D). However, by 20 dpf, 1% EtOH–exposed (12 hpf–5 dpf) larvae had a larger PVAT and smaller AVAT as a percentage of total VAT than control siblings (Figure 5, G–J). These findings indicate that EAE larvae have a preexisting propensity for altered lipid storage that may be reflective of a permanent alteration in lipid handling.

We next determined whether diet-induced obesity risk was present immediately after ethanol exposure or whether it developed later during life. Both 0% and 1% EtOH–exposed (12 hpf–5 dpf) AB larvae were subjected to a normal or a HFHC diet, which was proven to significantly increase VAT area and volume in WT larvae, and subsequently stained with Nile red for VAT size quantification (Supplemental Figure 5, E–H). Following ND, no significant difference in VAT size was observed between control and EAE cohorts; however, with HFHC diet, EAE larvae developed a 60.3% larger VAT volume/body length ratio (*P* = 0.0096) and a 40.7% larger VAT area/body area ratio (*P* = 0.0005) relative to controls (Figure 5, K and L, and Supplemental Figure 5, I and J). The significantly enlarged VAT size in HFHC diet–fed EAE larvae was initially achieved at 13 dpf (Supplemental Figure 5K). Enlarged VAT volume in EAE larvae resulted from an increased number of adipocytes. Although VAT cell number was significantly reduced relative to controls in EAE larvae receiving the ND (*P* = 0.0345), HFHC diet caused the EAE cohort to develop significantly more visceral adipocytes than sibling-matched controls (*P* = 0.0410) (Figure 5M). Both control and EAE larvae exhibited HFHC diet–induced adipocyte hypertrophy; however, no significant difference in cell diameter was observed between control and EAE fish under normal or HFHC diet conditions (Supplemental Figure 5, L and M). These data demonstrate that EAE changes the response to an HFHC diet to enhance VAT gain via an increase in adipocyte number and indicate that alterations in adipocyte development followed by compensatory growth set EAE larvae up for diet-induced obesity as early as 13 dpf.

### EAE induces lasting multiorgan gene expression changes, which respond uniquely to HFHC diet.

**Figure 6:**
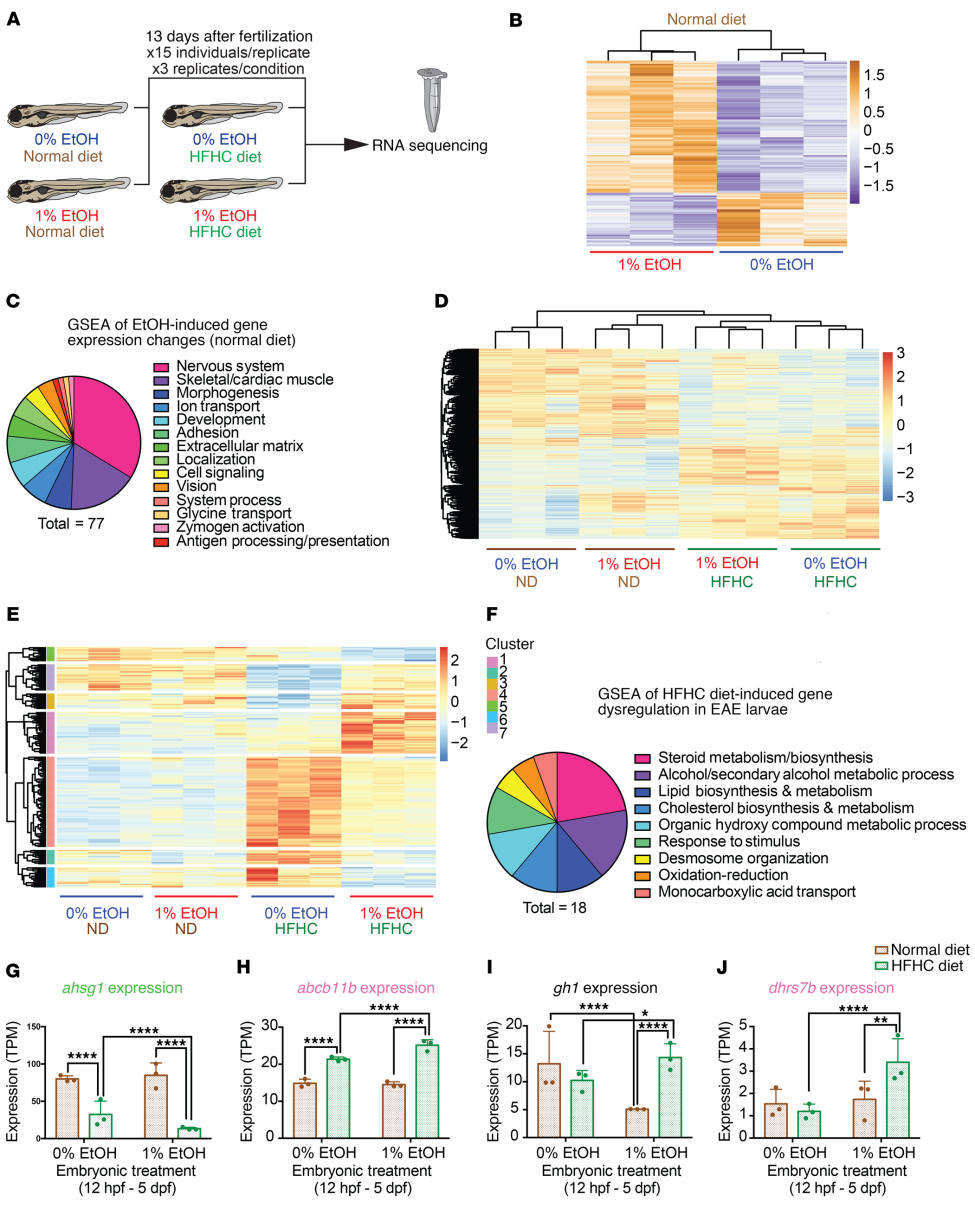
RNA-Seq identifies genes that interact with ethanol and diet to shape larval phenotypes. (**A**) Schematic of larval RNA-Seq. (**B**) Under ND conditions, EAE induces alterations in gene expression (*P_adj_* < 0.05). (**C**) GSEA identifies dysregulated pathways in 1% EtOH larvae receiving the ND. (**D**) HFHC diet challenge shifts the gene expression profile of both 0% and 1% EtOH–exposed (12 hpf–5 dpf) larvae (*P_adj_* < 0.05). (**E**) Cluster analysis of genes that are altered in 1% EtOH–exposed larvae in response to HFHC diet but not ND. (**F**) GSEA of HFHC diet-responsive genes from the 1% EtOH–exposed larval cohort. (**G**–**J**) Diet modulates gene expression in EAE larvae. Affected genes include those expressed in the liver (*ashg1*, *abcb11b)*, the brain (*gh1)*, and globally (*dhrs7b*). (**G** and **H**) ****P < 0.0001. (**I**) ****P < 0.0001; *P = 0.032. (**J**) ****P < 0.0001; **P = 0.004. *P* values were determined using a negative binomial test with a Wald’s test from RNA-Seq analysis.

Developmental alterations often result from perturbations in transcriptional programs. In order to decipher the molecular mechanisms underlying altered diet-induced VAT gains in EAE animals, we performed bulk RNA-Seq on pooled control and EAE AB strain larvae in the presence and absence of HFHC diet (Figure 6A). After recovering 8 days from EtOH exposure, 13 dpf EAE larvae receiving ND had 864 transcriptional changes that met the significance threshold of *P_adj_* < 0.05 (Figure 6B and Supplemental Table 1). We categorized these differentially regulated genes using Gene Ontology enRIchment anaLysis and visuaLizAtion tool (GOrilla) Gene Ontology (GO) term analysis (Figure 6C and Supplemental Table 2). Alterations in the expression of genes critical for neuron projection guidance (*P* = 2.23 × 10^–08^), synaptic transmission (*P* = 4.76 × 10^–08^), and nervous system development (*P* = 3.11 × 10^–06^) were the most prevalent, suggesting that aberrations in neuronal function promote behavioral alterations and diet-induced obesity (Supplemental Table 2). These lasting transcriptional changes pinpoint regulators of FASD-related phenotypes and showcase the widespread and persistent effects that EtOH has on development.

The introduction of an HFHC diet altered gene expression profiles in both control and EAE larvae (Figure 6D and Supplemental Tables 3 and 4). In control cohorts, HFHC diet reliably induced transcriptional changes in genes previously associated with metabolism, obesity, fatty liver, diabetes, inflammation, and altered circadian rhythm (Supplemental Figure 6, A–C, and Supplemental Tables 3 and 5). Since HFHC diet, but not ND, increased VAT size in EAE larvae, we hypothesized that EAE larvae would have a unique transcriptional response to diet challenge. We first identified 1468 significant (*P* < 0.05) differences in gene expression between control and EAE larvae receiving the HFHC diet (Supplemental Tables 6 and 7). Of these significant changes, 130 genes had a log_2_(FC) of less than –0.5 and 135 had a log_2_(FC) of more than 0.5. Next, we performed unsupervised hierarchical clustering on significantly dysregulated genes using pheatmap. We discovered 7 clusters of genes whose expression levels in EAE larvae were more dramatically dysregulated following the introduction of the HFHC diet than during the ND (Figure 6E and Supplemental Table 8). Among these clusters, there was enrichment for categories related to steroid (*P* = 9.48 × 10^–09^) and lipid biosynthesis (*P* = 1.5 × 10^–06^), cholesterol metabolic processes (*P* = 6.13 × 10^–07^), and secondary alcohol metabolic processes (*P* = 3.35 × 10^–07^; Figure 6F and Supplemental Table 9). These pathways were primarily dysregulated in EAE larvae in response to HFHC diet challenge, demonstrating that, in addition to the baseline changes induced by EAE, there is a unique molecular response of EAE larvae to HFHC diet. We also identified 4 clusters of genes that were differentially expressed in EAE larvae with ND, but not HFHC diet (Supplemental Figure 6D). Cluster 1 genes, which were downregulated in EAE larvae receiving the ND, exhibited a partial rescue in expression with HFHC diet (Supplemental Figure 6, E and F, and Supplemental Tables 10 and 11). Taken together, these findings demonstrate that EAE can interact with diet to influence gene expression in the developing larvae.

Of the disease-relevant genes that were differentially regulated in EAE larvae, several were significantly HFHC diet responsive. Orthologs of fetuin-A (*ahsg1*) and ATP binding cassette subfamily B member 11 (*abcb11b)*, liver-expressed genes associated with obesity and metabolic syndrome, were downregulated and upregulated in response to HFHC diet, respectively; however, this transcriptional response was significantly greater in EAE larvae (Figure 6, G and H, and refs. 47–50). Genes directly associated with adipogenesis and obesity, including growth hormone 1 (*gh1)* and dehydrogenase/reductase 7 *(dhrs7b)*, were also uniquely dysregulated in EAE larvae in response to HFHC diet (Figure 6, I and J, and refs. 51–53). Although no gene appears to singlehandedly explain the propensity for diet-induced VAT gain in EAE individuals, shifts in the expression of genes relevant to metabolism and lipid handling suggest that a suite of transcriptional changes likely contributes to this phenotype.

### EAE impairs hepatic development and increases the propensity for hepatic stress.

**Figure 7:**
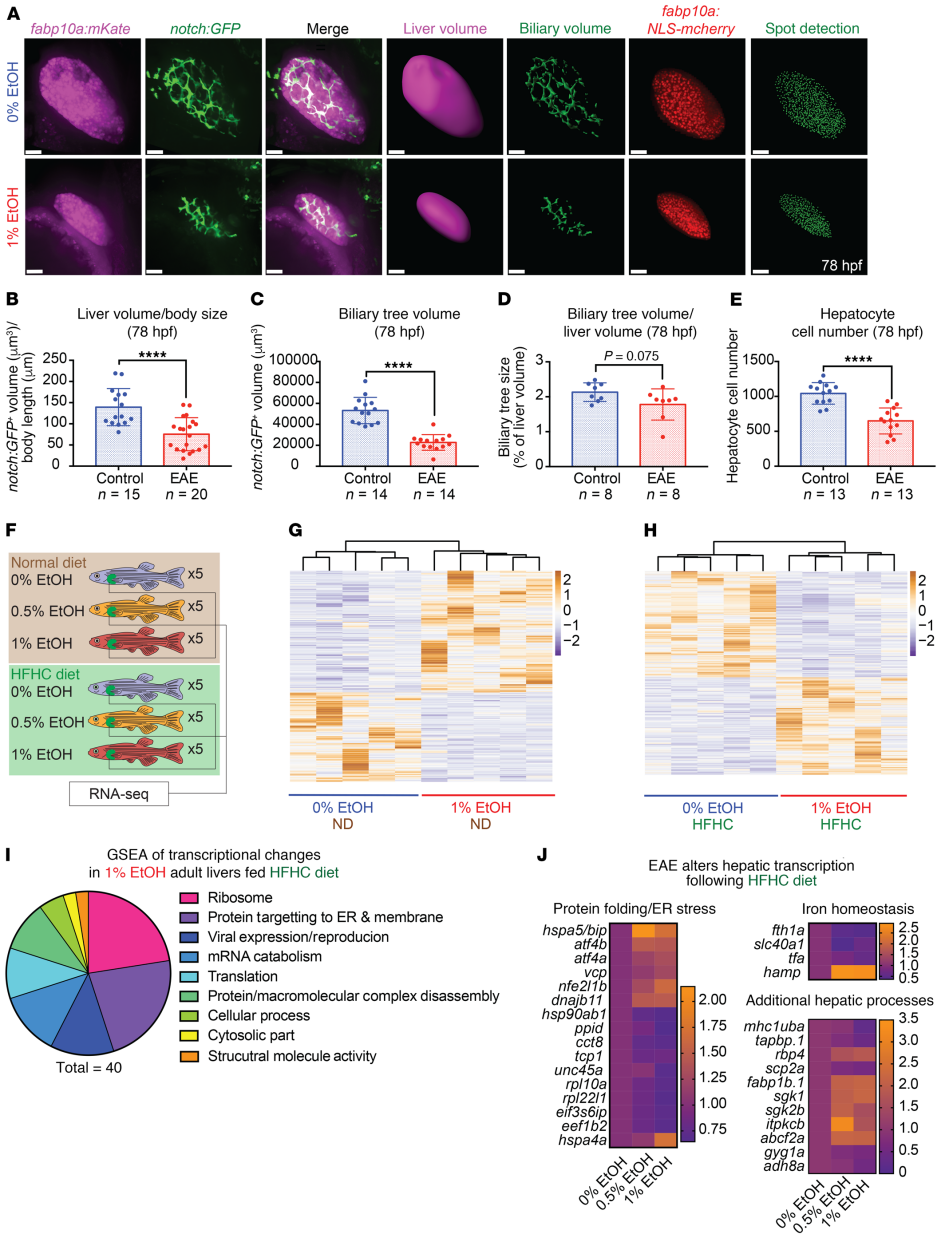
Ethanol impairs embryonic liver growth and alters liver response to HFHC diet. (**A**) EtOH (12 hpf–78 hpf) reduces the size of the *fabp10a:mKate^+^* liver and the size and complexity of the *tp1glob:eGFP^+^* biliary tree at 78 hpf. Liver and biliary volumes were achieved through 3D reconstruction based on confocal imaging of the *Tg(fabp10a:mKate^+^)* and *Tg*(*tp1glob:eGFP)* reporters. Confocal imaging and Imaris 3D spot detection of nuclei in *Tg(fabp10a:NLS-mcherry)* embryos demonstrate that EtOH exposure reduces hepatocyte nuclei number. Scale bars: 60 μm. (**B**) EAE reduces liver volume relative to body size. ****P < 0.0001, unpaired 2-tailed Student’s *t* test. (**C** and **D**) EAE reduces biliary tree volume, but proportionally to liver volume reduction. ****P < 0.0001, unpaired 2-tailed Student’s *t* test. (**E**) Hepatocyte number is reduced in 1% EtOH larvae relative to matched controls. ****P < 0.0001, unpaired 2-tailed Student’s *t* test. (**F**) Schematic of RNA-Seq of adult livers after 8 weeks of normal and HFHC diet challenge. (**G** and **H**) Heatmap of dysregulated (*P* < 0.05) genes following sequencing of 0% and 1% EtOH (12 hpf–5 dpf) adults receiving ND and HFHC diet. (**I**) GSEA of genes significantly dysregulated (*P* < 0.05) in 1% EtOH (12 hpf–5 dpf) adults receiving the HFHC diet. (**J**) Alterations in hepatic transcripts following HFHC diet challenge (*P* < 0.05). Heatmap *P* values were determined using a negative binomial test with Wald’s test from RNA-Seq analysis. Sample numbers (*n*) noted under figure panels.

The liver is the primary metabolic organ of the body, and alterations in its response to HFHC diet are a risk factor for the development of metabolic syndrome (54). Therefore, we examined the impact of EAE on hepatic development using fluorescent reporter lines and in situ hybridization. EAE resulted in significantly reduced liver and biliary tree size, as visualized and quantified in *Tg(fabp10a:mKate)* and *Tg(tp1glob:eGFP)* transgenic lines at 78 hpf, and diminished the number of *Tg(fabp10a:NLS-mcherry)*^+^ hepatocytes (Figure 7, A–E). Outgrowth of the *foxa3^+^* hepatic and pancreatic buds was disrupted by EAE at 48 hpf, though hepatic progenitor specification was not substantially impaired (Supplemental Figure 7A). These data demonstrate that EAE impairs hepatic differentiation and outgrowth and indicate that altered development may contribute to metabolic abnormalities later in life.

Though control and EAE larvae exhibited similar rates of diet-induced hepatic steatosis following HFHC diet challenge, lasting transcriptional changes in EAE larvae led us to hypothesize that the transcriptional status and physiological function of the adult liver were perturbed following EAE (Supplemental Figure 7, B–D). To evaluate this possibility, we performed RNA-Seq in 5 individual livers from family-matched 0%, 0.5%, and 1% EtOH–exposed (12 hpf–5 dpf) AB strain cohorts following 8 weeks of normal and HFHC diet (30 individual livers) when BMI had normalized between the groups (Figure 7F). EAE (1% EtOH) livers from under ND conditions had 848 significant (*P* < 0.05) transcriptional alterations, but few (*n* = 10) met the significance threshold of *P_adj_* < 0.05. In contrast, EAE (1% EtOH) fish fed a HFHC diet had 96 dysregulated transcripts meeting the threshold of *P_adj_* < 0.05 (Figure 7, G and H, and Supplemental Table 12 and 13). GO term analysis identified core pathways in EAE (0.5; 1.0% EtOH) livers that were disrupted by HFHC diet (Figure 7I and Supplemental Tables 14 and 15). Translational termination (*P* = 2.37 × 10^–16^) and elongation (*P* = 4.05 × 10^–14^), the cytosolic ribosome (*P* = 1.81 × 10^–15^), and protein targeting to the ER (*P* = 1.12 × 10^–13^) were among the most significantly disrupted processes (Supplemental Tables 14 and 15).

In both 0.5% and 1.0% EtOH–exposed (12 hpf–5 dpf) cohorts, dysregulation of stress response and protein-folding genes, including genes indicative of ER stress (*bip/hspa5*, *atf4*, *vcp*, *nfe2l1b/nrf1)*, was evident (Figure 7J and Supplemental Tables 13 and 16). There was a significant increase in *atf4b* (*P* = 3.02E-05, 1% EtOH) and *bip* (*P* = 0.01, 1% EtOH), ER stress sensor genes whose expression was similarly elevated in response to tunicamycin-induced protein misfolding and ER stress by quantitative reverse-transcriptase PCR (qRT-PCR) in adult livers (Supplemental Figure 7, E and F, and Supplemental Tables 13 and 16). Heightened cell stress may serve as an indicator of more advanced hepatic dysfunction. In addition to cellular stress response genes, there was an upregulation of retinol binding protein 4 *(rbp4*) (*P* = 0.009, 1% EtOH*)*, a circulating factor that regulates glucose and lipid metabolism, in both 0.5% and 1% EtOH adults (Figure 7J, Supplemental Tables 13 and 16, refs. 55–57). Additional significantly dysregulated genes included those critical for immune function (*mhc1uba*, *P* = 6.71 × 10^–11^, 1% EtOH*)*, glycogen storage *(gyg1a*, *P* × 10^–07^, 1% EtOH*)*, and iron homeostasis *(fth1a*, *slc40a1*, *tfa*, *hamp)* (Figure 7J and Supplemental Tables 13 and 16). Taken together, these data reveal that multiple hepatic processes are dysregulated in EAE adults following the introduction of a HFHC diet, especially cell stress–response pathways, and that hepatic organ dysfunction may contribute to metabolic dysfunction in EAE adults.

## Discussion

The adult health outcomes associated with FASDs and the molecular mediators of FASD-related organ dysfunction remain largely unidentified (12). Here, we describe one of the first and largest adult human and zebrafish cohort studies to examine metabolic health outcomes in FASD. We demonstrate that patients with FASDs have short stature, an increased incidence of T2DM, lower HDL cholesterol, and elevated triglyceride levels relative to matched controls. The incidence of multiple metabolic abnormalities is markedly higher in males with FASDs despite a lower BMI. Additionally, female patients with FASDs have an increased risk of overweight and obesity. In the FASD zebrafish model, we confirm that EAE accelerates the progression toward certain features of the metabolic syndrome, including visceral adiposity, elevated BMI, and fasting hyperglycemia. We present several explanations for the acceleration to metabolic abnormalities in the zebrafish cohort, including reduced activity level, abnormal organ development, and a unique transcriptional response to diet challenge. Our data reveal that PAE is associated with adult metabolic aberrations, even in the absence of obesity, and that there may be value in evaluating for FASD and considering PAE as part of the clinical assessment for metabolic disease risk.

Higher obesity rates in FASD patients have previously been ascribed to the many documented alterations in eating behavior (58–60). EAE had no impact on adult food consumption in our zebrafish model. However, neurological transcripts were critical targets of EtOH exposure during development and there were accompanying reductions in adult activity level. We propose that mild reductions in activity level translate to reduced energy expenditure in this model, therefore compounding any existing metabolic propensity for weight gain. An additional likely cause of increased metabolic disease following EAE is small for gestational age phenotypes and associated compensatory growth. In zebrafish, EAE promoted growth restriction and affected adipocyte and hepatic development during stages of differentiation and outgrowth. EAE larvae experienced dramatic compensatory growth, which culminated in appropriate stature and the rapid recovery of VAT size via an increase in adipocyte cell number. The molecular mechanisms resulting in growth restriction and catch-up growth several days after EtOH removal remain unclear; however, notable alterations in growth dynamics in the zebrafish model complement previous studies that show that growth restriction followed by catch-up growth may be a critical determinant of metabolic perturbation later in life (61–68).

In addition to changes in growth rate, EAE zebrafish displayed unique whole-body and organ-specific transcriptional alterations, especially in response to dietary challenge. This indicates that there may be permanent metabolic and molecular differences in how FASD fish manage nutrient intake. Importantly, EAE larvae had a conspicuous misexpression of genes that affect lipid handling or absorption, metabolism, and obesity risk (51–53, 69, 70). Some of these transcripts were indicative of altered liver development, and we identified the liver as a critical target organ of EAE during development. Disruption of hepatic differentiation and outgrowth likely contributes to lasting detrimental impacts of EAE on metabolism and hepatocyte function. For example, fetal growth restriction has been shown to promote the development of nonalcoholic fatty liver disease (NAFLD) by enhancing the hepatic ER stress response (71–73). Consistent with these findings, HFHC diet–fed EAE males had a transcriptional signature consistent with hepatic dysfunction that included indications of a chronic stress response and translational repression. Translational repression is a frequent consequence of the unfolded protein response (UPR), which often occurs in NAFLD (74–76). Chronic ER stress indicates a more progressed fatty liver state and suggests that there are intrinsic differences in the ability of EAE livers to respond to dietary stress. Future studies should examine the role of identified dysregulated genes in promoting specific metabolic health outcomes, as some may be druggable targets.

Many metabolic abnormalities in the FASD human cohort were also present in zebrafish, including obesity and altered glucose homeostasis, suggesting that pathophysiological mechanisms following EAE are conserved. However, one important distinction between the male human and zebrafish cohorts is that EAE zebrafish displayed diet-induced obesity, whereas the FASD patients had metabolic abnormalities in the absence of obesity. There are likely a number of reasons for these differences, not least of which are the interspecies differences in growth, adipostasis, and BMI sensing. The data collected in the human study were limited by its retrospective design, and therefore the human cohort likely contains a broad range of EtOH exposure, with variation in disease severity, in contrast with our zebrafish cohorts; severity of the FASD diagnosis could affect obesity and metabolic disease risk in adults. Future prospective studies in patients with FASDs, which may include careful collection of phenotypic data as well as data on resting energy expenditure and caloric intake, could help better delineate the observed differences and may provide greater insight into factors that contribute to the metabolic abnormalities in FASD patients.

An important question raised by the human data is whether diet-induced hyperglycemia and hepatic stress in EAE zebrafish is obesity dependent. One indication that metabolic abnormalities in zebrafish may be somewhat independent of obesity is that EAE fish challenged with HFHC diet quickly reached the same BMI as their control siblings. At the point at which male EAE zebrafish displayed fasting hyperglycemia and transcriptional changes in the liver, they had the same visceral adiposity and BMI as controls. However, it is well documented that an increased duration of obesity increases the risk for T2DM (77). EAE zebrafish have a more rapid progression toward central obesity and therefore experience excess central adiposity for a greater duration of time. This may accelerate diet-induced glucose and hepatic dysregulation. Further studies are needed to clarify obesity-dependent and obesity-independent metabolic abnormalities in FASDs.

In conclusion, our study identifies a common fetal stressor to be causal for lifelong alterations in metabolic health. Most immediately, our study will provide physicians and society in general with information that enables them to more fully understand the diverse potential risks associated with PAE, to preemptively monitor metabolic health parameters in at-risk patients, and to consider the implementation of measures to prevent metabolic disease. Importantly, this study expands our mechanistic understanding of the target organs of PAE and how organ dysfunction may alter an individual’s lifelong risk for disease. We define adipocytes and the fetal liver, organs rarely considered in the context of FASD, as new critical target tissues of EAE. Finally, we identify whole-larvae and organ-specific transcriptional changes that are diet responsive and that likely contribute to FASD phenotypes. Bringing to light potential molecular targets enables us to begin the discussion of what additional research is needed to enable identification of the critical regulators and target them therapeutically.

## Methods

### Human cohort.

We used the patient database registry at a large academic health system (RPDR) to identify the cohort of patients with a diagnosis of FASD. Using a medical record search query, we identified males and females 18 years of age or older with the diagnosis of FASD (*n* = 208) and a set of controls matched for age, sex, and race/ethnicity (*n* = 208). Statistical analysis was performed using JMP Pro 13.0 (SAS Institute) software. Means and SEM measurements are reported and were compared using the 2-tailed Student’s *t* test unless the data were nonnormally distributed, in which case medians and interquartile range were compared using Wilcoxon’s test. Categorical variables were compared using Pearson’s χ^2^ test. Least-squares linear regression modeling (for continuous dependent variables) or logistic regression (for binary dependent variables) was performed to control for relevant covariates. A *P* value of less than 0.05 on a 2-tailed test was used to indicate statistical significance.

### Animal studies.

The AB strain was used for all population studies, diet challenges, and RNA-Seq experiments. *Tg(fabp10a:mKate)*, *Tg(tp1glob:eGFP)*, and *Tg(fabp10a:NLS:mcherry)* lines from a mixed TU/AB/TL background were used to examine liver and biliary development (78–80). For all studies, clutch-matched siblings were randomly assigned to each treatment group. When BG, adiposity, liver size, or transcriptional changes were evaluated, only clutch-matched siblings were compared. All cohort studies included equal numbers of fish from contributing clutches. For all larval, juvenile, and adult pilot experiments, fish were used without sex bias. Male-specific phenotypes were evaluated in male cohorts as indicated.

### Chemical exposure.

Zebrafish larvae were exposed to 0%, 0.5%, and 1.0% EtOH dissolved in fish water from 12 hpf to 5 dpf. For all longitudinal studies, fish were removed from ethanol at 5 dpf and transferred to system water. Adults were exposed to 0.25 μg/mL to 1.0 μg/mL tunicamycin for 24 hours in system water. Livers from tunicamycin-treated (Sigma-Aldrich, T7765) animals were dissected from euthanized fish and harvested for RNA at the completion of the 24-hour treatment.

### Zebrafish diet challenge.

Larval zebrafish were housed on the nursery in 0.8-L tanks at a density of 32 fish per tank. At 5 dpf, fish received a paramecia starter culture and were kept overnight without flow. At 6 dpf, flow was initiated, and larvae were administered a specified diet. For larval obesity studies, larvae received either a ND consisting of hatched artemia 2 times per day at a density of approximately 8.8 mg dry weight/fish or a HFHC diet of artemia plus a dietary supplement of 0.5 g/tank of Hoosier Hill Farm Whole Egg Powder resuspended in system water. Upon graduation from the nursery (30 dpf), juvenile fish were maintained at equal tank densities (15–20 individuals, depending on the study) and fed a standard artemia diet 2 times per day at a density of approximately 14.7 mg dry weight/fish. Beginning at 60 to 65 dpf, fish were housed at a density of 12 fish/tank in 2.8-L (confirmatory study) or 6-L tanks (pilot study) and fed a normal or HFHC diet. For the 1.8-L tank diet challenge, fish were housed at a density of 10 fish/tank. Adult ND consisted of 0.013 g/fish of Skretting Gemma Micro 300 feed per day (59% protein, 14% lipids, 14% ash, 8% moisture, 0.2% crude fiber, and 1.9% starch by mass). HFHC diet tanks received 0.063 g/fish of Skretting Gemma Micro 300 per day plus 0.02 g/fish of Hoosier Hill Farm Whole Egg Powder (~49% lipids, ~49% protein, ~1.7% cholesterol). The resulting HFHC diet amounted to approximately 56.6% protein, 22.4% lipids, 0.41% cholesterol, 10.62% ash, 0.15% fiber by mass.

### Body measurements.

Zebrafish were fasted overnight and anesthetized in 0.4 mg/mL Tricaine solution. Juvenile and adult fish were measured using a standard ruler, weighed on an analytical balance, and transferred to a recovery tank. BMI was computed as weight/length^2^. BG concentrations were obtained after a 24-hour fast. Fish were euthanized in ice water, and blood was obtained through tail removal followed by centrifugation into heparin-lithium–coated tubes (81, 82). Glucose measurements were performed using a Bayer Contour Blood Glucose Monitoring System and associated test strips.

### Analysis of adult population study.

We modeled the effect of diet and ethanol exposure on BMI using a linear model. Our response variable (*BMIΔ_i_*) for fish (i) was the BMI of each fish at any given week minus the mean BMI of the corresponding tank in the previous week. Using linear regression in R, we included diet as a categorical variable and percentage of ethanol exposure as a quantitative variable. We also included the interaction between both variables. This model corresponds to the following: *BMIΔ_i_* *=* *μ* *+* *β_diet_diet_i_* *+β_ethanol_ethanol_i_* *+* *β*_diet × ethanol_*diet_i_* *×* *ethanol_i_* *+* ∈*_i_* where μ is the intercept, *β_diet_*, *β_ethanol_*, and *β_diet × ethanol_* are the corresponding coefficients that we infer using linear regression, and ∈ is the noise term. In R, we used the command lm(BMI_change ~ 1 + diet + ethanol + diet:ethanol). Since fish came from 4 different genetically distinct families, we tested the effect of genetic background by including family ID as a random effect. We tested each full mixed model to the corresponding nested model without the family ID using an F test and concluded that including family ID did not improve the model in a statistically significant way.

### Nile red and oil red O stain.

In order to visualize SAT and VAT, fish were fasted overnight and stained with Nile red (Invitrogen, Thermo Fisher Scientific, N1142). Fish were submerged in 0.5 μg/mL (from a 1.25 mg/mL acetone stock solution) Nile red for 30 minutes and washed in clean system water for 15 minutes. To visualize SAT, adults were submerged in 10 mg/mL epinephrine in fish water for 5 minutes (45). Before imaging and dissection, fish were euthanized in ice water. Nile red–stained zebrafish larvae were examined either with a Zeiss Discovery V8 stereoscope for wide-field imaging or with a Zeiss LSM 880 confocal microscope using a 10×/0.3 NA EC Plan-Neofluar objective lens. For wide-field imaging, Nile red fluorescence was analyzed with a 470 ± 20 nm excitation filter and 525 ± 25 nm emission filter. For confocal imaging, Nile red fluorescence was excited using 514 nm laser light, and the emission was collected using a 539 to 753 nm range. Oil red O stain was used to evaluate hepatosteatosis as previously described (83).

### VAT quantification.

VAT and SAT area were calculated using the area function in FIJI. The Imaris software (Bitplane) surfaces function was used to quantify the volume of larval fat droplets from confocal images. 3D volumetric renderings were constructed around Nile red^+^ visceral adipocytes, and total volume measurements were extracted. Adipocyte diameter was calculated by measuring the diameter of the individual lipid droplets visible in the confocal stack. VAT area and volume were normalized to animal length or animal body area.

### Histology and adult adipocyte diameter calculation.

Adult zebrafish at 0, 4, and 8 weeks of diet challenge were fixed in 10% NBF or Dietrich’s solution, paraffin embedded, serially sectioned, and stained with H&E at HistoWiz. The diameter of individual pancreatic visceral adipocytes was measured and averaged along the full length of the pancreas.

### Food consumption assay.

Individual zebrafish were housed off flow in breeding tanks for 10 days and administered Topfin Betta Bits mini floating food pellets at indicated intervals. The total number of pellets consumed per individual was converted to a food weight and normalized to body weight.

### Activity assays.

For locomotor assays, adult zebrafish were fed 1 hour before study initiation and placed in a clear 2.8-L system tank suspended above a white background. The day before solo locomotor assays, zebrafish were introduced to the assay tank for 5 minutes. On the day of the trial, fish were habituated for 10 minutes before recording. Average speed (cm/s) was calculated for each individual over 30-second and 3-second bins as indicated. For 1-minute novel tank assays, individual zebrafish were placed in a clear 2.8-L novel system tank suspended above a white background and habituated for 1 minute. Swimming activity was recorded for 1 minute, and fish were returned to their original tank. Fish were filmed in an isolated room with the videographer absent. Analysis of fish behavior was performed using Actualtrack Software (Actual Analytics).

### Modified Blazka-type swim chamber testing.

Adult AB zebrafish with prior 0% or 1% EtOH exposure (12 hpf–5 dpf) were subject to a modified acrylic Blazka-type swim chamber (350 mm l × 47 mm w × 90 mm d) with a flow-through rate of approximately 5.5 L/min^–1^. System water was introduced into 1 side of the swim chamber and funneled through a honeycombed grid composed of 50 tubes (6.35 mm 0D, 3.97 mm ID × 50 mm l). This created a laminar flow through the remaining 0.6-L swimming area (250 mm l × 50 mm d × 47 mm w). Fish were removed from their holding tanks and placed inside the flowing swim chamber for testing. A high-definition Nikon D3100 digital camera recorded swimming performance at 30 frames per second for a total of 5 minutes. The raw digital file was analyzed for movement within the swim area. The distance from the flow source and rear baffle was quantified using MATLAB (MathWorks).

### RNA isolation for RNA-Seq.

RNA was extracted in TRIzol (Life Technologies) from whole pooled (15 individuals/sample) 13 dpf larvae or single livers isolated from WT (AB) fish and purified using the QIAGEN RNeasy Mini Kit. RNA quality was verified on the Agilent Bioanalyzer, and DNA contamination was removed with the TURBO DNA-*free* (Invitrogen, Thermo Fisher Scientific) kit. Single-end NextSeq Series high-output RNA-Seq was performed on poly(A) selected coding mRNAs at the Dana-Farber Center for Cancer Computational Biology.

### RNA-Seq and gene set enrichment analysis.

For larval RNA-Seq (13 dpf), reads were aligned to the GRCz10 reference assembly with the STAR aligner and differential gene expression was performed with DESeq2 software using a negative binomial with Wald’s test. Gene set enrichment analysis (GSEA) was performed using the GOrilla GO enrichment analysis and visualization tool and the DAVID Bioinformatics Resources 6.8 Analysis Wizard (84–86). Heatmap visualization and hierarchical clustering were performed using the R package pheatmap with the default parameters. For the adult liver RNA-Seq, FASTQ files were analyzed using FASTQC to ensure uniform read quality (phred > 30) (87). Single-end reads were aligned using Star, version 2.3, to the zebrafish genome (GRCz10) (88). The mapped reads were counted using htseq-count (version 0.6.0, parameters –t exon) and gene models from Ensembl transcriptome v89 (89). Analysis of differential gene expression was performed using DESeq2 (90). GO term enrichment analysis was performed using Gage package and normalized counts (91). Significant enrichment of gene sets was identified using *P* < 0.001. GSEA was performed using a preranked running mode and ranking genes by log_2_(FC) (92).

### qPRT-PCR.

cDNA libraries were synthesized from TRIzol/chloroform-isolated RNA using the Bio-Rad iScript cDNA Synthesis Kit (catalog 1708891). RT-PCR reactions were performed using the iScript RT Supermix for RT-PCR (catalog 1708841). For qRT-PCR, the following primers were used: *bip* (forward: 5′-ATCAGATCTGGCCAAAATGC-3′; reverse: 5′-CCACGTATGACGGAGTGATG-3′), *ef1α* (forward: 5′-GCGTCATCAAGAGCGTTGAG-3′; reverse: 5′-TTGGAACGGTGTGATTGAGG-3′). Relative expression levels were calculated using the ΔΔCt method. Expression was normalized to *ef1α*.

### Fluorescence determination of liver size.

Confocal images of control and EAE embryos were obtained after overnight fixation in 4% PFA. To evaluate liver size, *Tg(fabp10a:mKate)* and *Tg(fapb10a:NLS-mcherry)* lines were examined; for biliary tree structure and size, *Tg(tp1glob:eGFP)* lines were employed (78–80). Confocal stacks were uploaded and 3D reconstructions were generated using Imaris software. To calculate liver cell number, the total number of nuclear dots in the *Tg*(*fabp10a:NLS-mcherry)* were counted using Imaris software 3D spot detection.

### In situ hybridization.

In situ hybridization was conducted according to standard protocols using established probes (93, 94).

### Statistics.

Relevant statistical analyses are described in the corresponding Methods subsection and identified in the figure legend. For multiple comparisons within a single diet group, 1-way ANOVA or unpaired 2-tailed Student’s *t* test was used. For multiple comparisons spanning diet or treatment conditions, 2-way ANOVA with multiple comparisons was used. When performing unpaired *t* tests, an F test was used to compare variances. If assumptions of the statistical test were not met, a nonparametric test was used. Population analysis was conducted using linear regression. Outliers were identified with ROUT (Q = 1%). Larval and adult RNA-Seq data presented in this study were deposited in the NCBI’s Gene Expression Omnibus database (GEO GSE142311).

### Study approval.

All animal studies were approved by the Institutional Animal Care and Use Committees at the Beth Israel Deaconess Medical Center (IACUC-BIDMC 056-2015) and the Brigham and Women’s Hospital (2016N000405), Boston, Massachusetts, USA. The Partners HealthCare institutional review board approved the human study (2017P000752), and informed consent was waived.

## Author contributions

OW, WG, MEC, PKF, and MLS conceptualized the work. OW, WG, PKF, and MLS developed methodology. OW validated results. OW, PKF, MLS, GDB, SA, IMO, YH, RTP, and ST engaged in formal analysis. OW, PJW, KL, AS, DK, II, IA, ST, and AT investigated. OW, WG, PKF, and MLS wrote the original draft. OW, PJW, and IMO were responsible for visualization. WG, PKF, and MLS supervised. OW and WG administered the project. WG and OW acquired funding.

## Supplementary Material

Supplemental data

Supplemental Tables 1-16

## Figures and Tables

**Table 1:**
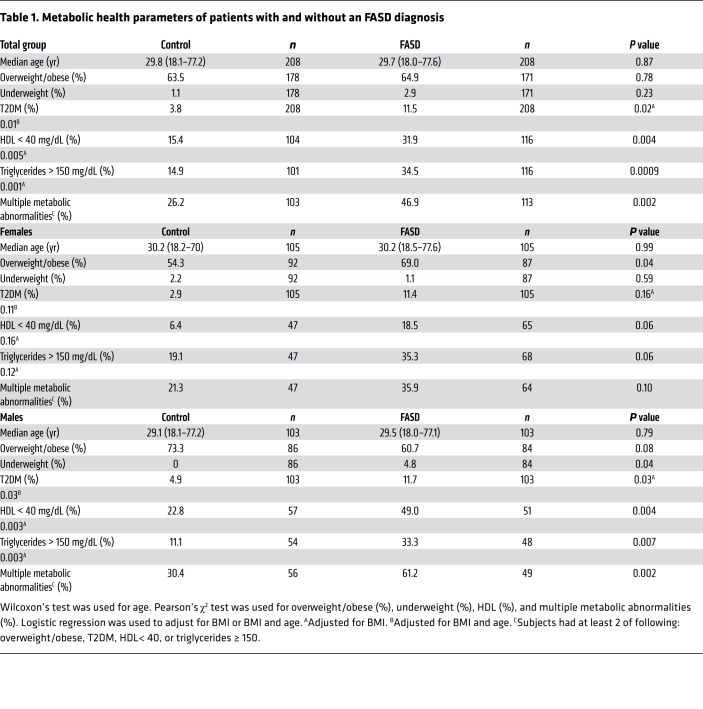
Metabolic health parameters of patients with and without an FASD diagnosis

## Version 1 03/23/2020

Electronic publication

## Version 2 05/01/2020

Print issue publication
