# Supplementary material for: Fetal alcohol spectrum disorder predisposes to metabolic abnormalities in adulthood
Source: J Clin Invest. 2020 Mar 23;130(5):2252–69. doi: 10.1172/JCI132139 (PMC7190939; doi:10.1172/JCI132139)
Supplement: Supplemental data [file jci-130-132139-s079.pdf]

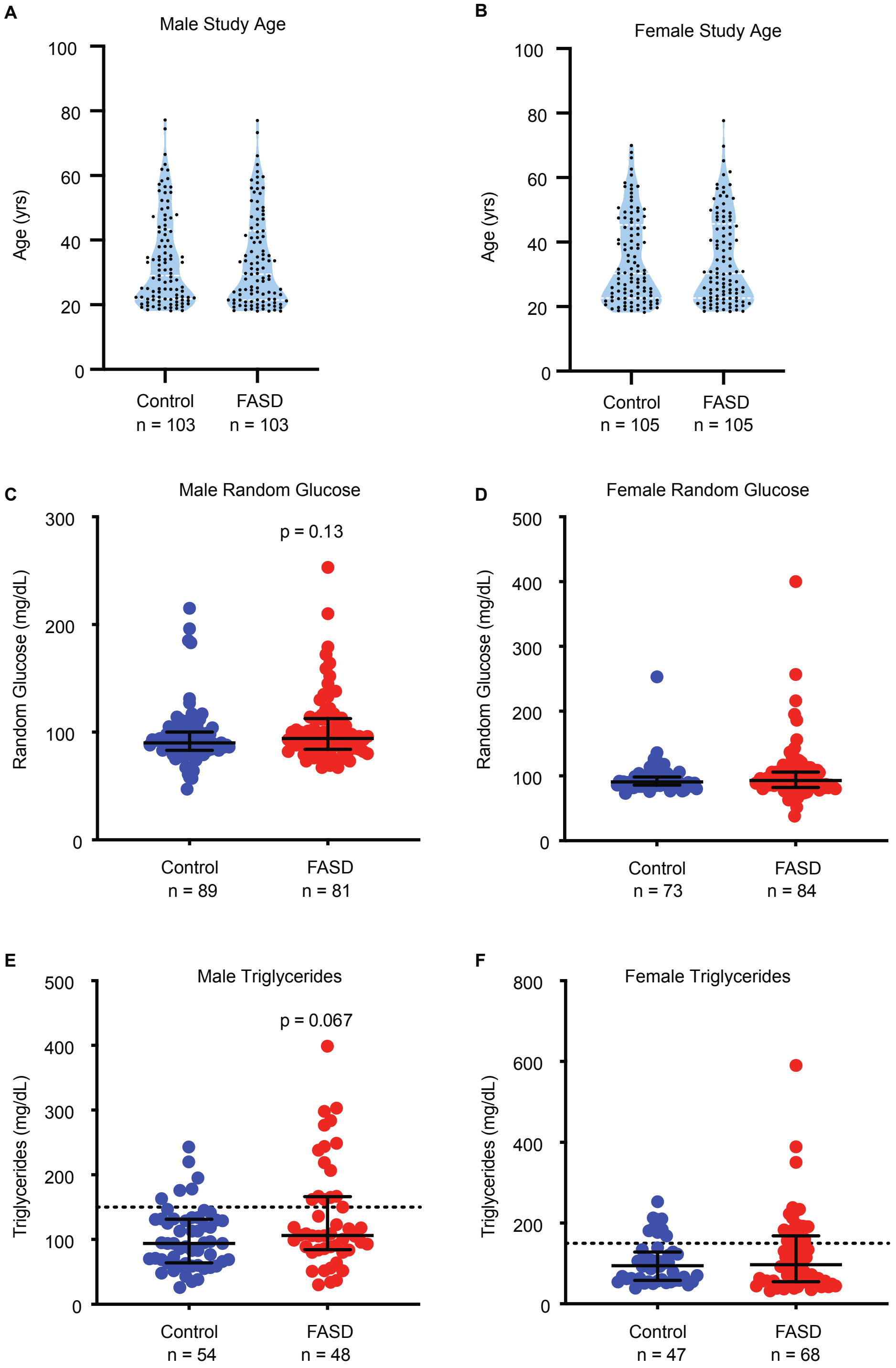


**Figure S1. Metabolic health parameters in the FASD human cohort. A – B.** Age distribution of control and FASD cohorts. **C – D.** Random blood glucose (RBGL) measurements in the male (p = 0.13)[1] and female (p = 0.98)[1] cohort. **E – F.** Triglyceride levels in the male (p = 0.067)[1] and female (p = 0.75)[1] cohort. [1]Wilcoxon test. Sample numbers (n) noted under figure panels. Error bars show median with interquartile range.

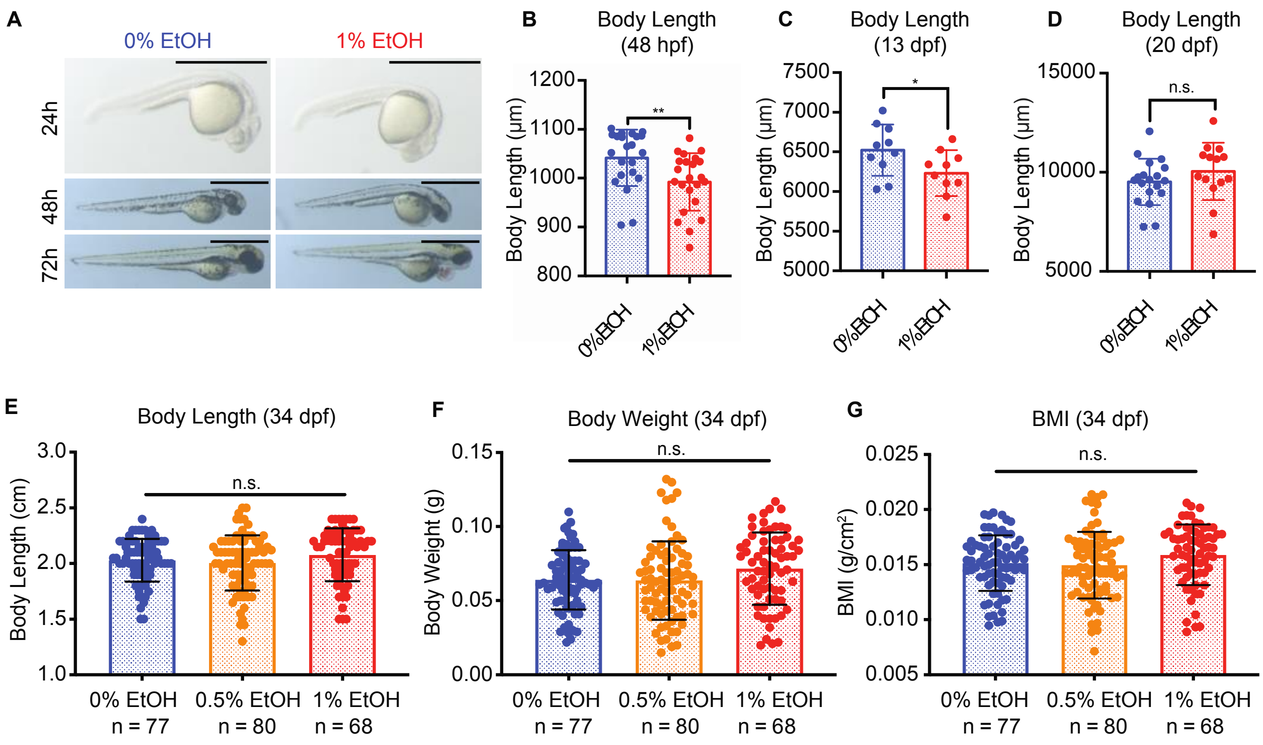


**Figure S2. Body parameters in larvae and juvenile zebrafish following EAE.**

**A.** Impact of EAE on development from 48 hpf – 72 hpf. Scale: 1mm. **B – D.** EAE reduces body length at 48 hpf through 13 dpf. Catch-up growth restores body length by 20 dpf. **E – G.** EAE exposure did not impact body length, weight, or BMI at 34 dpf prior to sexual differentiation. Sample numbers (n) noted under figure panels. Error bars show mean with SD.

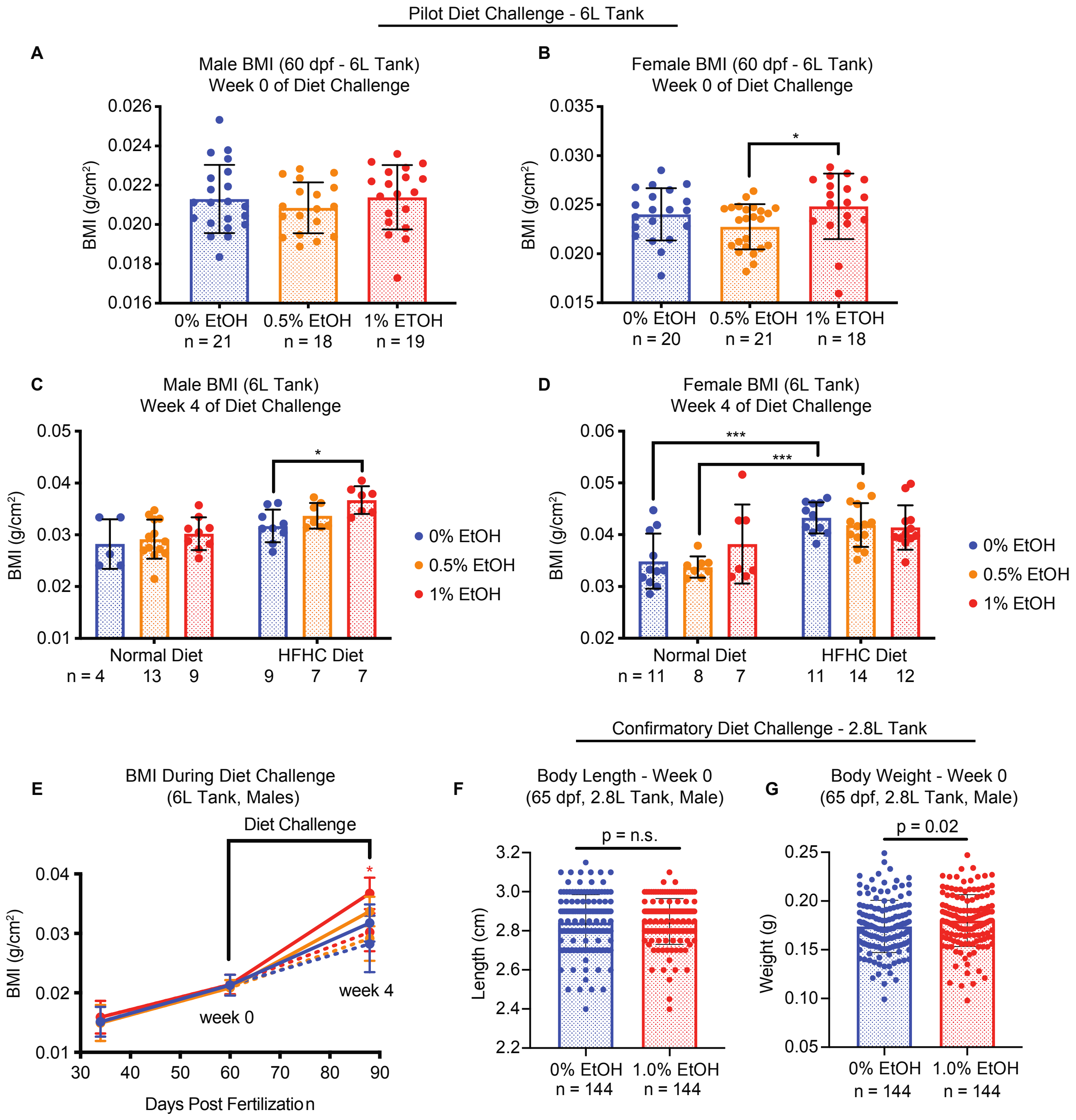


**Figure S3. Embryonic alcohol exposure is a risk factor for elevated BMI in males.**

**A – B.** BMI at 60 dpf in males and females enrolled in the pilot diet challenge study (6L tanks). No significant differences in BMI are evident in males. In females, the 1% EtOH (12 hpf – 5 dpf) cohort has a larger BMI than the 0.5% EtOH (12 hpf – 5 dpf) cohort ($p < 0.05$, one-way ANOVA with Tukey's multiple comparisons test). **C – D.** BMI after 4 weeks of the normal and HFHC trial diet challenge. 1% EtOH-exposed (12 hpf – 5 dpf) males but not females have a higher BMI relative to controls in response to HFHC diet. (C: $p = 0.0144$, two-way ANOVA with Tukey's multiple comparisons test; D: $p = 0.0002$ (0% EtOH), $p = 0.0005$ (0.5% EtOH), two-way ANOVA with Sidak's multiple comparisons test). **E.** Trajectory of BMI gain over the duration of the pilot study ($p = 0.0144$, two-way ANOVA with Tukey's multiple comparisons test). **F – G.** Body

length and weight at 65 dpf for control and EAE fish housed in 2.8L tanks (G: $p = 0.02$, unpaired one-way t-test). Sample numbers (n) noted under figure panels. Error bars show mean with SD.

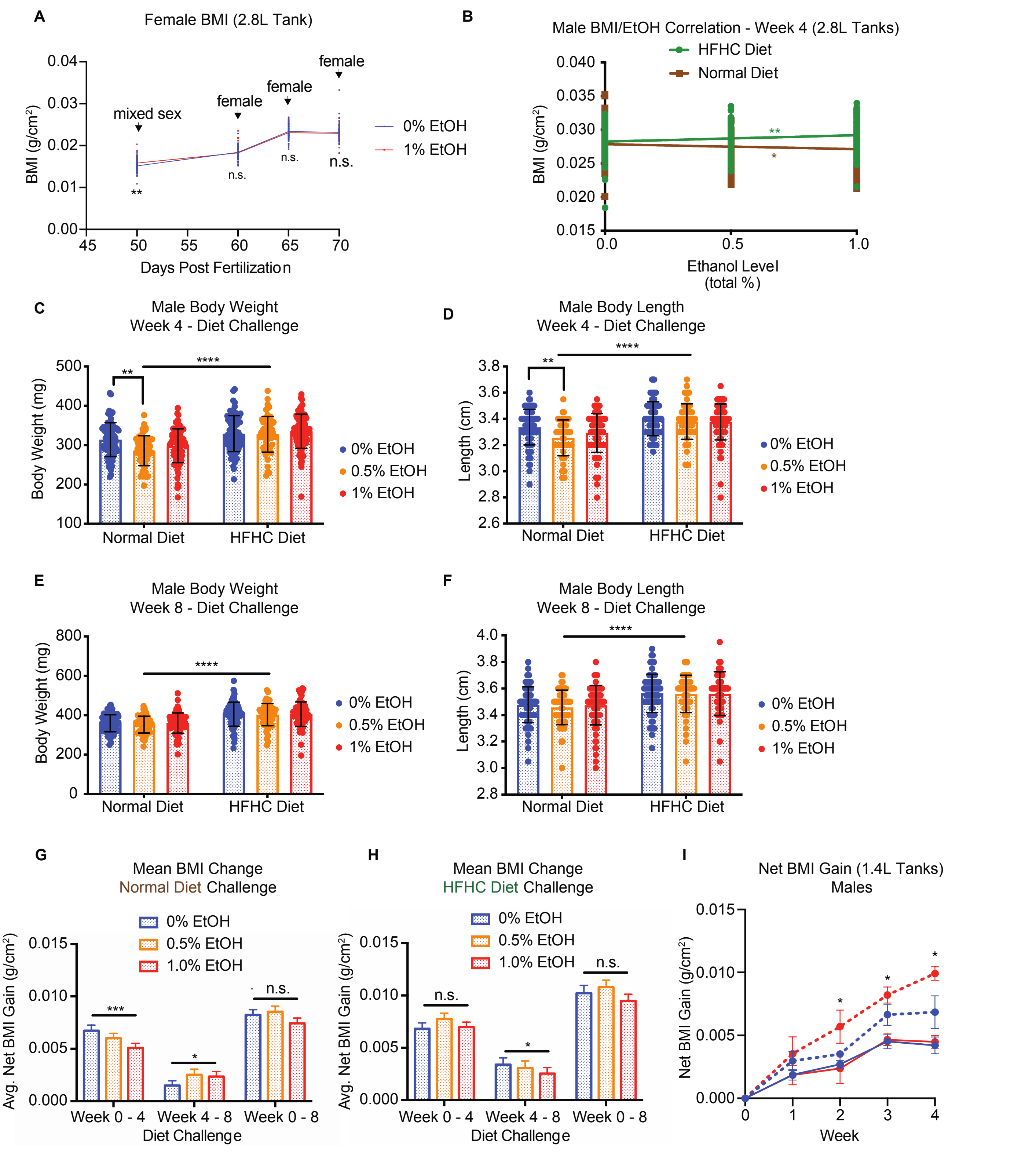


**Fig. S4. Body parameters in juvenile and adult zebrafish. A.** Female BMI during juvenile stages (**p = 0.001, unpaired two-tailed t-test; n> 30/group). **B.** Linear regression of BMI after 4 weeks of diet challenge (**p = 0.0074, *p = 0.0319). **C.** Body weight following 4 weeks of dietary challenge (**p = 0.0019, ****p < 0.0001, two-way ANOVA with Tukey's multiple comparisons test). **D.** Body length following 4 weeks of dietary challenge (**p = 0.0062, ****p <0.0001, two-way ANOVA

with Tukey's multiple comparisons test). For C – D, n = 84 (0% EtOH, ND), n = 60 (0.5% EtOH, ND), n = 84 (1% EtOH, ND), n = 78 (0% EtOH, HFHC), n = 60 (0.5% EtOH, HFHC), n = 84 (1% EtOH, HFHC). **E. – F.** Body weight and body length is impacted by diet but not EAE after 8 weeks of challenge (****p <0.0001, two-way ANOVA with Tukey's multiple comparisons test). For E – F, n = 83 (0% EtOH, ND and HFHC), n = 60 (0.5% EtOH, ND and HFHC), n = 84 (1% EtOH, ND and HFHC). **G.** EAE zebrafish receiving a normal diet gain less weight during weeks 0 – 4 and more BMI during weeks 4 – 8 compared to controls (Week 0 - 4: slope = -0.0016, p = 2.15e-06; Week 4 - 8: slope = 0.00084, p = 0.0255). **H.** EAE zebrafish receiving a HFHC diet gain less weight during weeks 4 – 8 compared to controls after having achieved a larger BMI than controls during earlier juvenile stages (Week 4 – 8: slope = -0.00087, p = 0.0225). P-values determined using linear regression. **I.** Net BMI gain in males housed in 1.4L tanks for 4 weeks of diet challenge beginning at 65 dpf (p < 0.05, unpaired one-tailed t-test, n = 3 tanks of 10 fish per group). Error bars show mean with SD.

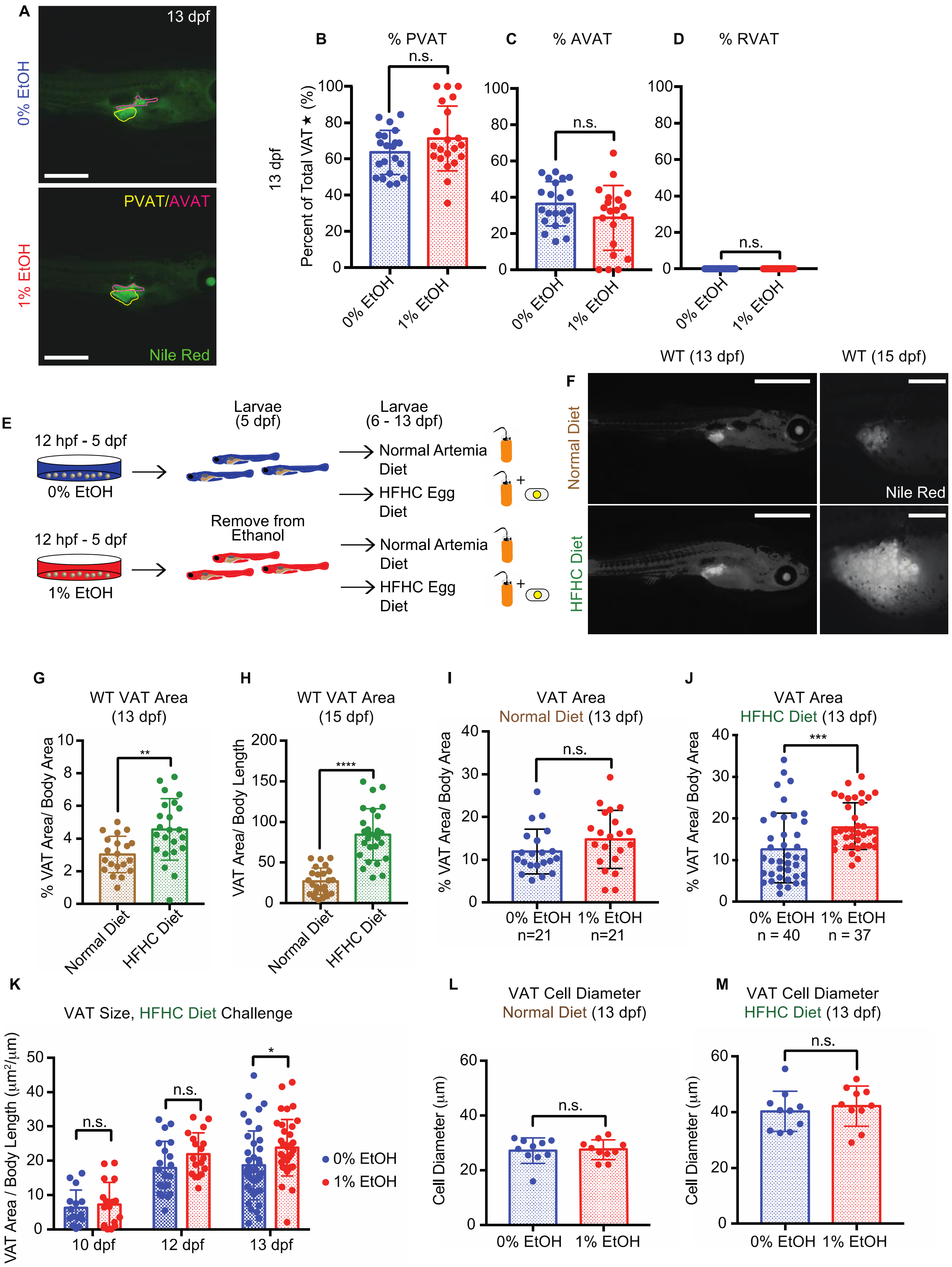
A
13 dpf
0% EtOH
PVAT/AVAT
1% EtOH
Nile Red
B
% PVAT
n.s.
13 dpf
Percent of Total VAT★ (%)
0% EtOH
1% EtOH
C
% AVAT
n.s.
D
% RVAT
n.s.
E
12 hpf - 5 dpf
0% EtOH
Larvae
(5 dpf)
Larvae
(6 - 13 dpf)
Normal Artemia
Diet
HFHC Egg
Diet
Remove from
Ethanol
12 hpf - 5 dpf
1% EtOH
F
WT (13 dpf)
WT (15 dpf)
Normal Diet
HFHC Diet
Nile Red
G
WT VAT Area
(13 dpf)
% VAT Area/ Body Area
Normal Diet
HFHC Diet
H
WT VAT Area
(15 dpf)
VAT Area/ Body Length
I
VAT Area
Normal Diet (13 dpf)
% VAT Area/ Body Area
0% EtOH
n=21
1% EtOH
n=21
J
VAT Area
HFHC Diet (13 dpf)
0% EtOH
n = 40
1% EtOH
n = 37
K
VAT Size, HFHC Diet Challenge
VAT Area / Body Length (µm²/µm)
10 dpf
12 dpf
13 dpf
0% EtOH
1% EtOH
L
VAT Cell Diameter
Normal Diet (13 dpf)
Cell Diameter (µm)
M
VAT Cell Diameter
HFHC Diet (13 dpf)

**Figure S5. Impact of EtOH and diet on VAT development. A.** Nile Red stained larvae at 13 dpf. PVAT and AVAT depots are highlighted in yellow and magenta, respectively. Scale: 1mm. **B – D.** No significant differences in % PVAT, % AVAT, or % RVAT are present at 13 dpf (n = 21 animals/group). **E.** Overview of larval HFHC diet challenge in control and EAE cohorts. **F.** Nile red stained VAT in 13 dpf and 15 dpf wildtype larvae. HFHC diet increases the size of the VAT depot, validating the efficacy of the diet. Scale: 1mm. **G – H.** Quantification of VAT area in 13 dpf and 15 dpf wildtype larvae (**p < 0.01; ****p <0.0001, two-tailed Mann-Whitney test). G: n = 20 (ND), n = 23 (HFHC); H: n = 24 (ND), n = 28 (HFHC). **I – J.** Percent VAT area relative to total body area in control and EAE larvae following normal and HFHC diet challenge at 13 dpf. VAT area/body area is increased in EAE larvae with HFHC diet challenge (p = 0.0005, two-tailed Mann-Whitney test). **K.** Time course of VAT size following HFHC diet challenge. 1% EtOH exposure promotes diet-induced VAT gains initially at 13 dpf (p = 0.0173, unpaired two-tailed t-test). For 10 dpf: n= 15 (0% EtOH), n = 17 (1% EtOH). For 12 dpf: n = 20 (0% EtOH), n = 18 (1% EtOH). For 13 dpf: n = 40 (0% EtOH), n = 37 (1% EtOH). **L – M.** Mean VAT cell diameter in control and EAE larvae following normal and HFHC diet at 13 dpf (n = 10 animals/group). Error bars show mean with SD.

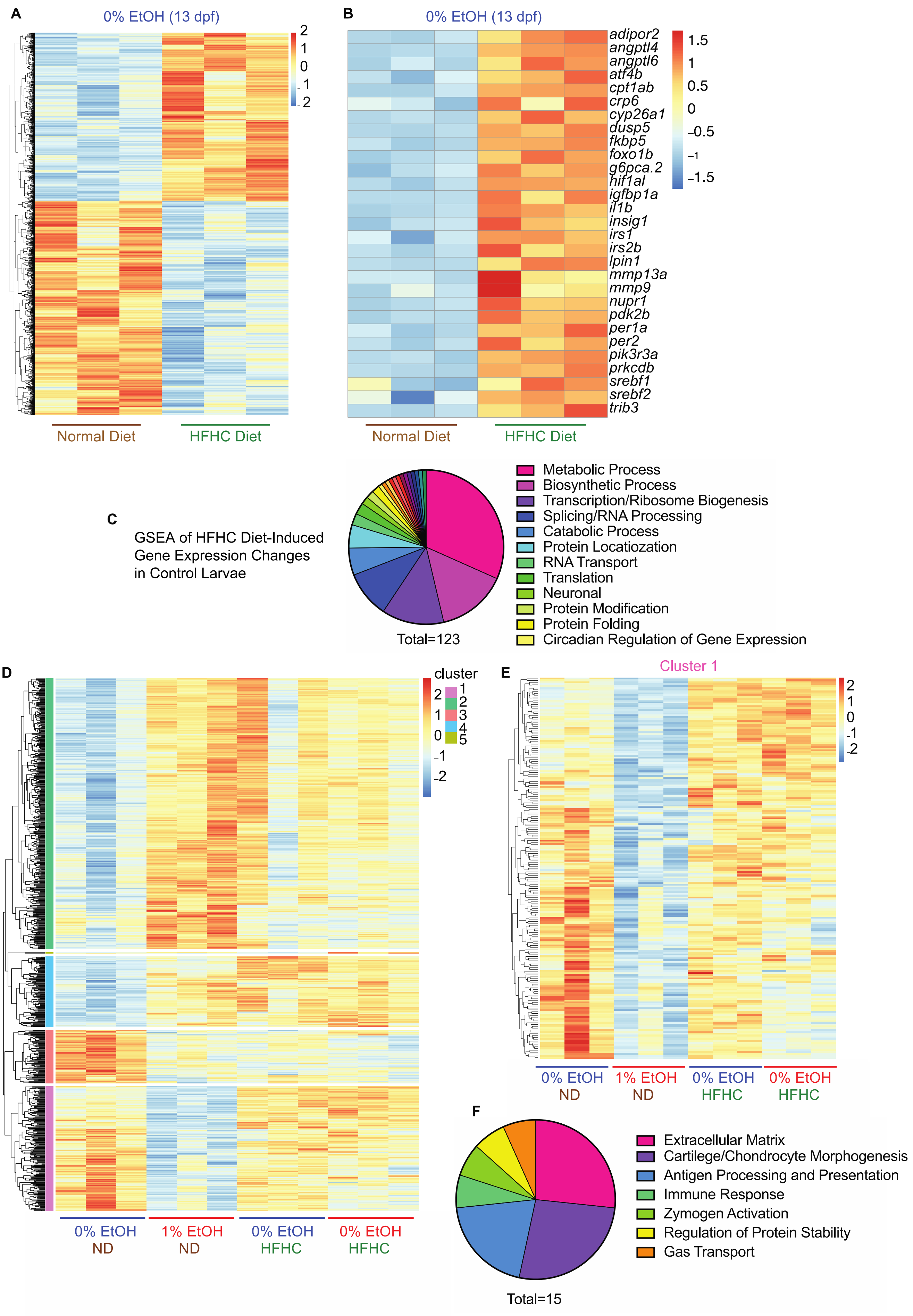
A
0% EtOH (13 dpf)
Normal Diet
HFHC Diet
B
0% EtOH (13 dpf)
adipor2
angptl4
angptl6
atf4b
cpt1ab
crp6
cyp26a1
dusp5
fkbp5
foxo1b
g6pca.2
hif1al
igfbp1a
il1b
insig1
irs1
irs2b
lpin1
mmp13a
mmp9
nupr1
pdk2b
per1a
per2
pik3r3a
prkcdb
srebf1
srebf2
trib3
Normal Diet
HFHC Diet
C
GSEA of HFHC Diet-Induced Gene Expression Changes in Control Larvae
Total=123
Metabolic Process
Biosynthetic Process
Transcription/Ribosome Biogenesis
Splicing/RNA Processing
Catabolic Process
Protein Locatiozation
RNA Transport
Translation
Neuronal
Protein Modification
Protein Folding
Circadian Regulation of Gene Expression
D
cluster
0% EtOH ND
1% EtOH ND
0% EtOH HFHC
0% EtOH HFHC
E
Cluster 1
0% EtOH ND
1% EtOH ND
0% EtOH HFHC
0% EtOH HFHC
F
Total=15
Extracellular Matrix
Cartilege/Chondrocyte Morphogenesis
Antigen Processing and Presentation
Immune Response
Zymogen Activation
Regulation of Protein Stability
Gas Transport

**Figure S6. GSEA and cluster analysis of differentially regulated genes identified in RNAseq. A.** Heatmap of significant (padj<0.05) whole-body transcriptional changes in 13 dpf control larvae in response to HFHC diet. **B.** Heatmap of significant (padj<0.05) obesity-related transcriptional changes in control larvae in response to HFHC diet. From top to bottom, genes include: *adipor2, angptl4, angptl6, aft4b, cpt1ab, crp6, cyp26a1, dusp5, fkpb5, foxo1b, g6pca.2, hif1al, igfbp1a, il1b, insig1, irs1, irs2b, lpin1, mmp13a, mmp9, nupr1, psk2b, per1a, per2, pik3r3a, prkcdb, srebf1, srebf2,* and *trib3.* **C.** GSEA of HFHC diet-induced transcriptional changes in 13 dpf control larvae identifies critical pathways normally disrupted by HFHC diet administration. **D.** Cluster analysis identifies genes that are more dysregulated in 1% EtOH-exposed larvae receiving the normal diet (relative to matched controls) than in the HFHC diet. **E.** Cluster 1 gene expression identifies transcripts downregulated in 1% EtOH-exposed larvae but partially rescued by HFHC diet. **F.** GSEA of transcripts identified in Cluster 1 pinpoints critical processes that are improved by HFHC diet. Heatmap p-values were determined using a negative binomial test with a Wald test from RNAseq analysis.

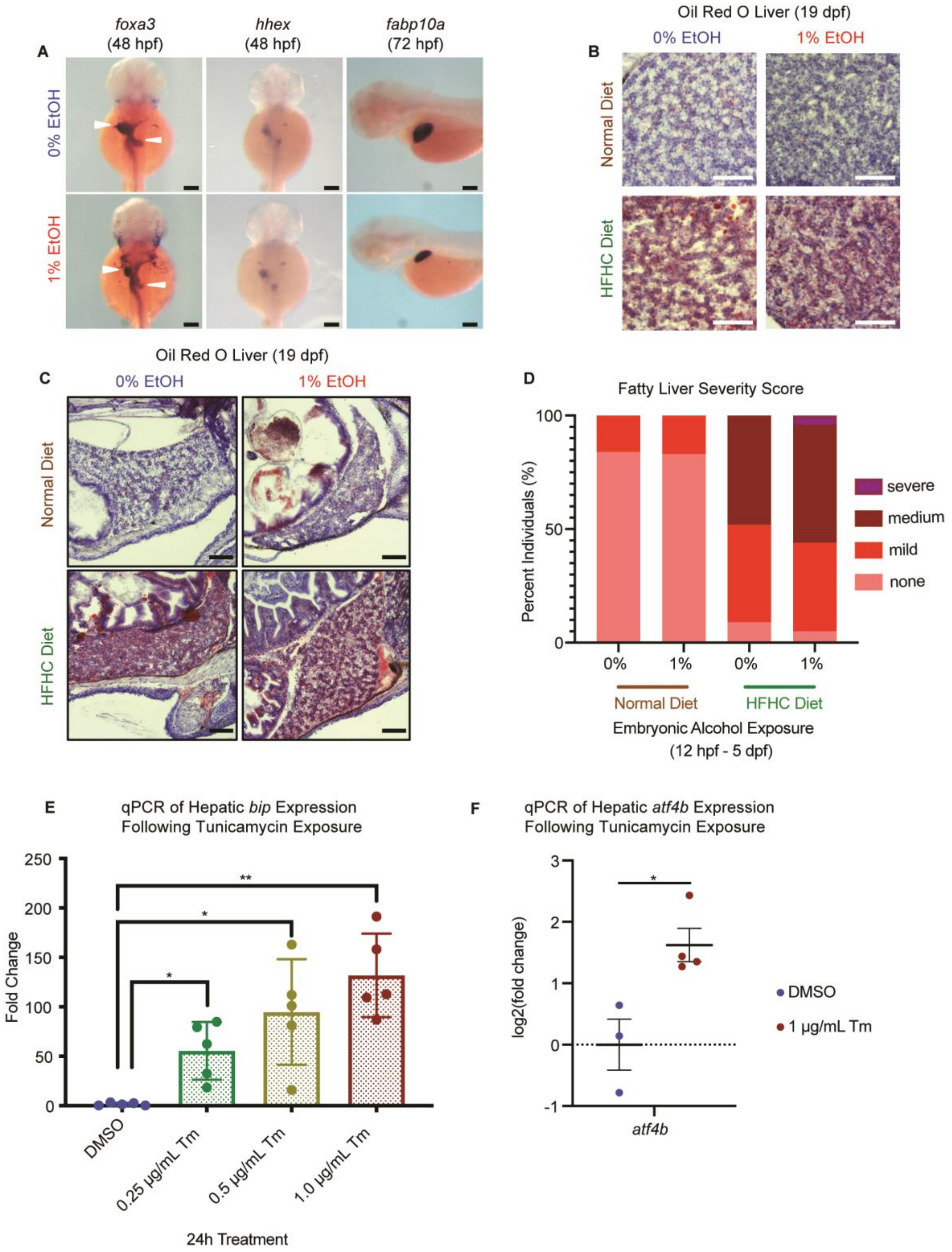
A
foxa3
(48 hpf)
hhex
(48 hpf)
fabp10a
(72 hpf)
0% EtOH
1% EtOH
B
Oil Red O Liver (19 dpf)
0% EtOH
1% EtOH
Normal Diet
HFHC Diet
C
Oil Red O Liver (19 dpf)
0% EtOH
1% EtOH
Normal Diet
HFHC Diet
D
Fatty Liver Severity Score
Percent Individuals (%)
100
50
0
severe
medium
mild
none
0%
1%
0%
1%
Normal Diet
HFHC Diet
Embryonic Alcohol Exposure
(12 hpf - 5 dpf)
E
qPCR of Hepatic bip Expression
Following Tunicamycin Exposure
Fold Change
250
200
150
100
50
0
**
*
*
DMSO
0.25 µg/mL Tm
0.5 µg/mL Tm
1.0 µg/mL Tm
24h Treatment
F
qPCR of Hepatic atf4b Expression
Following Tunicamycin Exposure
log2(fold change)
3
2
1
0
-1
*
atf4b
DMSO
1 µg/mL Tm

**Figure S7. The impact of EAE on liver biology. A.** *in situ* hybridization for pan endoderm marker *foxa3* and hepatic progenitor marker *hhex* at 48 hpf. Hepatic and pancreatic budding from the gut tube is delayed by 1% EtOH exposure (white arrows); however, 1% ETOH exposure does not prevent *hhex* hepatic progenitor specification. *in situ* hybridization for hepatocyte marker *fabp10a* at 72 hpf. 1% EtOH exposure delays the emergence of differentiated hepatocytes. **B – C.** Oil Red O staining of livers from 0% EtOH and 1% EtOH 19 dpf larvae. HFHC diet increases lipid levels in the liver and blood in control and EAE larvae. Scale: 0.01mm. **D.** Fatty liver severity score determined by lipid retention in larval hepatocytes. HFHC diet, but not prior EAE, induces more severe hepatic steatosis scores. For D: n = 19 (0% EtOH, ND); n = 23 (1% EtOH ND); n = 23 (0% EtOH HFHC); n = 23 (1% EtOH HFHC). **E.** qRT-PCR analysis of *bip* expression in response to 24h tunicamycin-induced ER stress in adult male livers (DMSO vs 0.25 µg/mL, p = 0.0363; DMSO vs. 0.5 µg/mL, p = 0.0439; DMSO vs 1ug/mL, p = 0.0059, Brown-Forsythe and Welch ANOVA test, Dunnett's T3 multiple comparisons test). Data represent mean with SD. n = 5 individuals per group. **F.** qRT-PCR of ER stress response genes *atf4b* in the presence and absence of tunicamycin (24 hpf) (p = 0.0187, two-tailed unpaired t-test). Data represent mean ± SEM. ER stress induces *atf4b* expression. n = 3 (DMSO), n = 4 (Tm).
